# Functional characterization of optic photoreception in *Lymnaea stagnalis*

**DOI:** 10.1371/journal.pone.0313407

**Published:** 2024-11-12

**Authors:** Alicia N. Harracksingh, Julia Bandura, Takefumi Morizumi, Philippe P. Monnier, Jeffrey T. Henderson, Zhong-Ping Feng

**Affiliations:** 1 Departments of Physiology, Temerty Faculty of Medicine, University of Toronto, Toronto, Ontario, Canada; 2 Department of Biochemistry, Temerty Faculty of Medicine, University of Toronto, Toronto, Ontario, Canada; 3 Department of Ophthalmology and Vision Sciences, Temerty Faculty of Medicine, University of Toronto, Toronto, Ontario, Canada; 4 Leslie Dan Faculty of Pharmacy, University of Toronto, Toronto, Ontario, Canada; Waseda University: Waseda Daigaku, JAPAN

## Abstract

Optic photoreception is a critical function for animal survival. Across the evolutionary spectrum, diverse animal models have been used to investigate visual system function and potential mechanisms under physiological or pathophysiological states. However less is known on photoreceptive behaviors and retinal processing in invertebrates, especially molluscs. This study focuses on the freshwater pond snail, *Lymnaea stagnalis* (*L*. *stagnalis*), to explore its visual function and underlying mechanisms. Using anatomical and histological approaches we characterized the *L*. *stagnalis* eye structure and demonstrated structural connections and retinal rhodopsin-positive sensory cells potentially critical for phototransduction. To assess the snail phototactic responses, we developed a new neurobehavioral protocol and employed DeepLabCut to track and quantify animal locomotion. We demonstrated that *L*. *stagnalis* exhibits a positive locomotory response to intense focal light and has diverse photo-locomotory responses. Further, we conducted phylogenetic and protein structure analyses and demonstrated that *L*. *stagnalis* has a unique repertoire of both vertebrate and invertebrate phototransduction genes. Further characterization of a rhodopsin-like gene identified unique characteristics compared to other mollusks and vertebrates, suggesting different mechanisms of phototransduction. Taken together, our work establishes *L*. *stagnalis* as a model organism for studying optic photoreception, offering new insights into the evolution and diversity of visual function across animal species.

## Introduction

The capacity for animals to react to light stimuli and integrate this sensory information is essential for survival. Despite the evolutionary diversification of sensory systems responsible for sight, locomotory movement as a response to input from the visual system has been a reliable measure of photoreceptive function [1]. Phototaxis, the movement response to light cues from the external environment [1], is a fundamental property of the visual system, typically driven by the activity of photoreceptor cells via rhodopsin [2]. In vertebrate and invertebrate models of vision, phototaxis is commonly used to assess the integration of visual-sensory signals in the central nervous system, typically seen in vertebrates such as rodents and zebrafish, and invertebrates, like annelids, arthropods, and mollusks [3].

Gastropod mollusks possess an intricate binocular visual system, however, express several light-sensitive photoreceptors at sites such as the eye, tentacles, and skin of the mantle (optic and dermal photoreceptors) [1, 4], unlike most vertebrates who possess just a single light-sensing organ (optic photoreceptors), integrated with photosensitive neurons in the central ganglion network [5, 6]. Mollusk binocular visual systems bear significant structural similarity to those seen in mammals [7], including a cornea, lens, multi-layered retina, and aggregate optic nerve [8] facilitating photoreception and driving phototaxis response [9–11]. The pond snail *Lymnaea stagnalis* (*L*. *stagnalis*) is of particular interest given its well-studied central nervous system (CNS), ability to be easily maintained in the laboratory, and breadth of literature on their behavioral response to a variety of stimuli. *L*. *stagnalis* eyes are clad with retinal photoreceptors proposed to be involved in light-sensitive behaviors and positive phototaxis, in which snails will actively extend their bodies to locomote toward a focal light source [12]. Additionally light-sensitive retinal photoreceptors in *L*. *stagnalis* eyes have been proposed to integrate sensory signals and regulate light sensitivity through first and second-order projections within the peripheral visual system, including the CNS [13, 14]. However, phototactic locomotory behavior and the underlying anatomical and molecular components in *L*. *stagnalis* remain unexplored.

Phototransduction is initiated in photoreceptors in which light of the appropriate wavelength ultimately triggers changes in photoreceptor membrane potential via isomerization of the 11-cis to trans retinal in GPCR-linked rhodopsin, promoting retinal release with subsequent activation of its downstream signaling cascade and ion channels. Means of phototransduction signalling are diverse throughout the animal kingdom in part due to rhodopsin’s interactions with different G-proteins present in vertebrates and invertebrates. In vertebrate rod photoreceptors, rhodopsins couple with the α-subunit of cytoplasmic G-protein transducin [15] and arrestin S-antigen (SAG) [16, 17] to initiate downstream cyclic nucleotide gated (CNG) ion channel opening [18]. By contrast most invertebrate rhodopsins couple with Gq-type G -proteins in rhabdomeric and ciliary photoreceptors [19, 20], typically resulting in activation pathways involving polyphosphoinositides and cyclic GMP (cGMP), ultimately triggering the activation of ion channels located within the photoreceptor plasma membrane [21]. Additionally, select molluscan rhodopsin homologs couple with Go-type G proteins [22], though the downstream molecules involved in this transduction process remain unclear. *L*. *stagnalis* bears a putative rhodopsin protein, as well as dermal photoreceptors bearing arrestin signatures [23] and cyclic nucleotide gated (CNG) ion channels [24], and retinal photoreceptors with suggested TRP channel-mediated retinal phototransduction through activation of inositol trisphosphate [25], though characterization of the rhodopsin with respect to its formal role in either the dermal or retina-based phototransduction pathways has yet to be performed.

In this study we sought to characterize the *L*. *stagnalis* visual system and explore the possibility of establishing an *in vivo L*. *stagnalis* model for analysing photoreception and phototaxis, using a machine learning-based approach. We anticipate that structural and functional descriptions of the *L*. *stagnalis* visual system allows for further characterization the molecular components potentially involved in the photoreceptive process and phylogenetic patterns of *L*. *stagnalis* rhodopsin and other key genes in this pathway. This study will establish *L*. *stagnali*s phototaxis as a novel *in vivo* model for assessing the fundamental mechanics of photoreception for the first time and offer insights into how the signalling pathway is conserved among gastropods.

## Materials and methods

### Animal care

Freshwater snails, *L*. *stagnalis*, were raised and kept at 20–22°C on a 12-hour light/dark cycle and fed green leaf lettuce and ground fish food flakes three times a week. Adult snails aged 2–3 months and 20–30 mm in length were used for this study.

### Tissue preparations, histology, immunohistochemistry, and electron microscopy

Adult animals were anesthetized in ‘snail saline’ (51.3mM NaCl, 1.7mM KCl, 4.1mM CaCl_2_, 1.5mM MgCl_2_, 2mM HEPES; pH 7.9) containing 10% Listerine. Once anesthetized, the mantle, including the eyes and tentacles, was dissected. For histology, tissue samples were fixed in 4% PFA for 16–18 hours, and then embedded in paraffin. The samples were serially sectioned at 7μm and mounted on charged microscope slides (VWR). After deparaffinization and rehydration, tissue slices were stained with either thionin or H&E following standard protocols, dehydrated, and digitally scanned using a Hamamatsu NanoZoomer 2.0 HT scanner (20x primary magnification) using NDP.view 2 software.

*For immunohistochemistry*, immunofluorescence labelling was performed on sections adjacent to those used for H&E or thionin staining. Following deparaffinization and rinsing in PBS, sections were pre-blocked using Background Sniper (BioCare Medical) and incubated for 18 hours at 4°C with anti-Octopus rhodopsin (Rabbit Anti- Octopus Rhodopsin (1:1000); CosmoBio Cat# LSL-LB-5509, RRID: AB_605170) overnight at 4°C. Following several washes in PBS containing 0.1% Triton-X 100 (EMD Millipore) (PBS-T), sections were incubated with anti-rabbit 488 antibody (Cell Signalling Technology) for 1 hr at room temperature. Sections were then counterstained with Hoescht 33258 (1 ug/mL; Cell Signalling Technology) for five minutes and washed with PBS-T several times before mounting in ProLong^™^ Gold Antifade Mountant (ThermoFisher Scientific). Fluorescence images were subsequently taken on a Confocal LSM700 Laser Scanning Microscope (Carl Zeiss).

*For electron microscopy*, samples were fixed in 2% paraformaldehyde, followed by 2.5% glutaraldehyde in 0.1M sodium cacodylate buffer for 2 hours each. Samples were then rinsed in buffer and post-fixed in 1% osmium tetroxide in buffer for 90 min, followed by dehydration in a graded series of ethanol baths (50%, 70%, 90% and 100%) for 20 minutes each step, and two propylene oxide changes for 30 min each. This was followed by embedding in Quetol-Spurr resin. Blocks were then cured overnight in the at 60°C. Seventy nanometer (70 nm) thick sections were then cut on an EM UC7 ultramicrotome (Leica) and post-stained with 2% uranyl acetate and 3% lead citrate for 20 minutes each and washed for 5 minutes after each staining. Sections were air dried at room temperature before viewing under TEM.

### Phototaxis arena design and behavioral testing

All behavioral experiments were performed at 20–22°C during the daylight component of the diurnal cycle on individual snails. Snails were transferred to a glass test chamber equipped with a black back panel, filled with approximately 5mm of pond water to prevent desiccation and reduce friction during gliding locomotory movements. Snails were acclimated to the test chamber for ~10 minutes before testing in standard room light (equal in intensity to the daylight portion of their cycle) and prior to being exposed to total darkness (dark phase) and focal light presentation (focal phase). At the start of each test snails were manually positioned at the ‘start’ (opposite to the location of the light source) in the phototaxis arena and placed there at the beginning of each subsequent testing phase (15 minutes/test) to track and quantify movements relative to the start position (Fig 4A, 4B). Video recording of snail locomotor movement during each testing phase was captured with a RaspberryPi Zero V1.1 recording single-board computer with camera module mounted to a goose neck holder positioned over the arena in the SD TV 576p 4:3 preset at a video resolution of 796 x 576 pixels, 25 recording and unboxing frames per second (fps) and at image resolution of 2592 x 1944 pixels. Videos thus consisted of ~ 22,500 frames containing both dark and focal phases. The RaspberryPi web-based interface (version 6.6.26) for controlling camera and video, including detection, time lapse, and recording capabilities were accessed vi. For full details regarding phototaxis arena design and testing are provided in the S1 File.

### Phototaxis analysis through DeepLabCut

For animal tracking and pose estimation of *L*. *stagnalis* phototaxis behavior, DeepLabCut (version 2.2.3; RRID:SCR_021391) [26] and Google Colaboratory were used following video acquisition (summarized in Fig 4C). DeepLabCut is an open-source software tool designed for animal pose estimation and behavior tracking from video data using deep neural networks learning. It tracks animal movements by non-invasively labeled body parts without markers and analyzes the movement and pose with high precision. Once trained, the network can process large amounts of video data automatically thus suitable for processing large datasets efficiently [26]. Briefly, the animal’s head, top edge of the shell closest to the head, bottom edge the shell furthest from the head, and shell apex were labelled (Fig 4D) in 50 frames taken from 10 videos of 10 different animals and then used in a ResNet-50-based neural network with default DeepLabCut parameters for 1024000 training iterations. Validation with 1 shuffle found the test error to be: 2.49 pixels, train error: 1.36 pixels. 5 outlier frames were then extracted from 100 videos and pose markers adjusted to refine the network. After merging original and refined datasets, the ResNet-50-based neural network was once again trained with default parameters for 1,000,000 iterations. Following refinement of the network, the test error was found to be: 1.66 pixels, train error: 1.38 pixels. The refined network was then used to analyze videos acquired under similar experimental settings. Analyzed predictions were filtered with a p-cut-off of 0.9 to generate final csv files containing each animal’s pose and XY position. Csv files were then analyzed in R (version 4.2.2; RStudio version 2022.7.2.576) using a custom script developed by the laboratory. Parameters were based on the position of the shell apex, as this body part was the most reliably detected by both the labeler and DeepLabCut.

Parameters examined included the total trajectory length in centimeters (determined using the TrajR package for R) and total trajectory speed in centimeters per second, latency to reach the region of the focal light, i.e., the focal light area, in minutes, and total time spent in the focal light area in minutes. To determine the focal light area, random unlabelled frames from the focal light phase of testing for all videos analyzed were extracted. Using ImageJ, the labeler drew a rectangular box (bounding box) for each frame capturing the focal light area, where the light from the focal light source was brightest within the recording chamber. The coordinates of this bounding box were extracted and used in subsequent analysis assessing response to focal light arena. To ensure consistency and prevent bias, the labeler completed their body part labelling and bounding box designations in a single-blind manner. For full details regarding the DeepLabCut processing, refer to S1 File.

### Bioinformatics and protein analysis

#### Comparative protein mining and modelling in *L*. *stagnalis*

Bioinformatic mining of select phototransduction KEGG pathway identities were done by first extracting the human or drosophila homologs of genes of interest in the phototransduction KEGG pathways for critical molecules involved in phototransduction. NCBI query sequences from the human phototransduction KEGG pathway (hsa04744) and *D*. *melanogaster* KEGG pathway (ko04745) can be found in Table 1. Top homologs of query sequences were searched for in the *L*. *stagnalis* transcriptome using BlastP (RRID:SCR_001010) against the *L*. *stagnalis* adult CNS next generation sequencing effort available at lymnaea.org [27], where the acceptable e-value cutoff was 1e-10. Top homolog hits were screens with SmartBlast and InterPro (RRID:SCR_006695) protein signature prediction software [28] to identify and confirm the final homolog identity. Transcript per million (TPM) data for mRNA of interest to the phototransduction pathway were obtained through recent *L*. *stagnalis* CNS transcriptome efforts from four independently sequenced CNS tissue samples and compared to reference genes. Beta-tubulin (TUBB), glyceraldehyde 3-phosphate dehydrogenase (GAPDH), and beta-actin (ACTB) were used in TPM analysis and mined from the transcriptome as reference genes [29].

**Table 1:** Human phototransduction KEGG pathway (hsa04744) molecular homologs used for *L*. *stagnalis* blastp query.

KEGG Pathway	Protein Identity	Protein Symbol	Query Species	Accesion number	Source	Lymnaea.org protein identifier (top hit)	L. stagnalis protein identifier (S1 Fig)
hsa04744	Rhodopsin	RHO	*Homo sapiens*	NP_000530	NCBI Reference Sequence	evgLocus_stringtie_AE_32017	RHO
	Calmodulin 1	CALM	*Homo sapiens*	NP_008819	NCBI Reference Sequence	evgLocus_Scallop_AE_9992	CALM1
		PDE6A	*Homo sapiens*	NP_000431	NCBI Reference Sequence	evgLocus_Trinity_AF_38508	PDE6A
	Recoverin	RCVRN	*Homo sapiens*	NP_002894	NCBI Reference Sequence	evgLocus_Scallop_AE_30170	RCVRN
	cyclic nucleotide gated channel subunit alpha 1	CNGA1	*Homo sapiens*	NP_000078	NCBI Reference Sequence	evgLocus_Trinity_AG_6913	CNGA1
	cyclic nucleotide gated channel subunit beta	CNGB1	*Homo sapiens*	NP_001288	NCBI Reference Sequence	evgLocus_Trinity_AG_2741	CNGB1
	S-antigen visual arrestin	SAG	*Homo sapiens*	NP_000532	NCBI Reference Sequence	evgLocus_Trinity_RF_Nov18_19466	SAG/ARR2
	G protein subunit alpha transducin 1	GNAT1	*Homo sapiens*	NP_000163.2	NCBI Reference Sequence	evgLocus_Trinity_GG_DRR_2657	GNAT1
	G protein subunit beta 1	GNB1	*Homo sapiens*	NP_001269468.1	NCBI Reference Sequence	evgLocus_strawberry_AE_24910	GNB1
	G protein subunit gamma transducin 1	GNGT1	*Homo sapiens*	NP_068774.1	NCBI Reference Sequence	evgLocus_Stringtie_DRR_50697	GNGT1
	G protein-coupled receptor kinase 1	GRK1	*Homo sapiens*	NP_002920.1	NCBI Reference Sequence	evgLocus_Trinity_AF_46266	GRK1
	Sodium/potassium-transporting ATPase subunit alpha-1	ATP1A1	*Homo sapiens*	P05023	UniProtKB/Swiss-Prot	evgLocus_Trinity_GG_DRR_21340	ATP1A1
ko04745	rhodopsin	RHO	*Homo sapiens*	P06002	UniProtKB/Swiss-Prot	evgLocus_stringtie_AE_32017	RHO
	guanine nucleotide-binding protein G(q) subunit alpha	GNAQ	*Homo sapiens*	P50148	UniProtKB/Swiss-Prot	evgLocus_strawberry_AE_32653	GNAQ
	guanine nucleotide-binding protein G(I)/G(S)/G(O) subunit gamma-13	GNG13	*Homo sapiens*	Q9P2W3	UniProtKB/Swiss-Prot	evgLocus_Stringtie_DRR_50697	GNG13
	phosphatidylinositol phospholipase C, beta	PLCB	*Drosophila melanogaster*	NP_476768.1	NCBI Reference Sequence	evgLocus_Trinity_AG_47545	PLCB
	transient receptor potential cation channel subfamily C member 4	TRPC4	*Homo sapiens*	Q9UBN4	UniProtKB/Swiss-Prot	evgLocus_Trinity_GG_DRR_1427	TRPC(A)
	transient-receptor-potential-like protein	TRPL	*Drosophila melanogaster*	P48994	UniProtKB/Swiss-Prot	evgLocus_Trinity_AE_48360	TRPC(B)
	inactivation no afterpotential D protein	INAD	*Drosophila melanogaster*	Q24008	UniProtKB/Swiss-Prot	N/A	N/A
	classical protein kinase C alpha type	PRKCA	*Homo sapiens*	P17252	UniProtKB/Swiss-Prot	evgLocus_strawberry_AF_26711	PRKCA
	Calmodulin	CALM	*Homo sapiens*	P0DP23	UniProtKB/Swiss-Prot	evgLocus_Scallop_AE_9992	CALM
	arrestin-2	ARR2	*Drosophila melanogaster*	P19107	UniProtKB/Swiss-Prot	evgLocus_Trinity_RF_Nov18_19466	SAG/ARR2
	inositol 1,4,5-triphosphate receptor type 1	ITPR1	*Mus musculus*	P11881	UniProtKB/Swiss-Prot	evgLocus_Trinity_GG_DRR_35036	ITPR1
	beta-adrenergic-receptor kinase	GRK	*Homo sapiens*	P25098	UniProtKB/Swiss-Prot	evgLocus_Trinity_AE_79470	ADRBK
	calcium/calmodulin-dependent protein kinase (CaM kinase) II	CAMK2	*Homo sapiens*	Q9UQM7	UniProtKB/Swiss-Prot	evgLocus_Trinity_AE_83463	CAMK2
	sn1-specific diacylglycerol lipase	DAGL	*Homo sapiens*	Q8NCG7	UniProtKB/Swiss-Prot	evgLocus_Scallop_AE_9329	DAGL
	serine/threonine-protein phosphatase with EF-hands	PPEF, PPP7C	*Drosophila melanogaster*	P40421	Drosophila melanogaster	evgLocus_Trinity_AF_55277	PPEF

For protein alignments depicted in this study, sequences were obtained first from the NCBI database, followed by GenBank as necessary. To obtain the rhodopsin homologs in animals of interest, NCBI BlastP analysis was conducted using the human rhodopsin (NP_000530) as query. The top blast hits from each search were screened sequentially against human rhodopsin for (1) completeness, using ClustalOmega (RRID:SCR_001591) pairwise alignment, (2) identity, using NCBI SmartBlast tool, and (3) rhodopsin domain topology, such as the presence of seven transmembrane bound helices and one cytoplasmic helix, using InterPro protein sequence analysis and classification server [29]. Confirmed, putative full-length rhodopsin sequences were subsequently aligned using the muscle alignment tool in the MEGA software (RRID:SCR_000667) version 10 [30], and then further analyzed in the JalView (RRID:SCR_006459) [31] for visualization. Alignments were colored using the ClustalX coloration tool, and analysed for sequence conservation and consensus sequence were included. Secondary structures (alpha helices, beta sheets, coils, etc) of aligned rhodopsin sequences were predicted using the PROMALS3D (SCR_018161) [32].

#### Phylogenetic inference

To generate the rhodopsin maximum-likelihood phylogenetic tree, protein or nucleotide sequences were obtained from the NCBI and GenBank databases for species spanning several metazoan animal phyla (Table 2). Where applicable, nucleotide sequences obtained for analysis were subsequently translated to protein coding sequences using ExPASY translate tool. Protein sequences were then aligned using the MUSCLE alignment tool in the MEGA version 10 software package (RRID:SCR_023471) [33]. The aligned sequences were then trimmed with Trimal v.1.3 (RRID:SCR_017334) [34] with ranging gap thresholds. The chosen trimmed alignment (gap threshold 0.7) was processed for phylogenetic model selection and maximum likelihood construction with 1000 ultrafast ultra-fast bootstraps using IQ-TREE web server (RRID:SCR_017254) [35]. Tree construction(s) were then analyzed with MEGA version 10, and coloration was done using Adobe Illustrator (RRID:SCR_010279).

**Table 2:** Sequence data for rhodopsin proteins use for generation of the maximum-likelihood phylogenetic tree.

Sequence	Phyla	Species	Accesion number	Source
*Homo sapiens* NP_000530.1 Rhodopsin	Chordata	*Homo sapiens*	NP_000530.1	NCBI Reference Sequence
*Bos taurus* NP_001014890.1 Rhodopsin	Chordata	*Bos taurus*	NP_001014890.1	NCBI Reference Sequence
*Xenopus tropicalis* OCT85772.1 Rhodopsin	Chordata	*Xenopus tropicalis*	OCT85772.1	GenBank
*Danio rerio* Q9W6A6.2 Rhodopsin	Chordata	*Danio rerio*	Q9W6A6.2	UniProtKB/Swiss-Prot
*Macrostomum lignano* PAA91957 Rhodopsin	Platyhelminthes	*Macrostomum lignano*	PAA91957.1	GenBank
*Schistosoma haematobium* CAH8679464 Rhodopsin	Platyhelminthes	*Schistosoma haematobium*	CAH8679464.1	GenBank
*Drosophila melanogaster* P06002 OPS1 Rhodopsin 1	Arthropoda	*Drosophila melanogaster*	P06002	UniProtKB/Swiss-Prot
*Drosophila melanogaster* NM_079674.3 Rhodopsin 2	Arthropoda	*Drosophila melanogaster*	NM_079674.3	NCBI Reference Sequence
*Drosophila melanogaster* NM_079687.3 Rhodopsin 3	Arthropoda	*Drosophila melanogaster*	NM_079687.3	NCBI Reference Sequence
*Drosophila melanogaster* NM_057353 Rhodopsin 4	Arthropoda	*Drosophila melanogaster*	NM_057353	NCBI Reference Sequence
*Drosophila melanogaster* NM_057748.5 Rhodopsin 5	Arthropoda	*Drosophila melanogaster*	NM_057748.5	NCBI Reference Sequence
*Drosophila melanogaster* NM_079644.3 Rhodopsin 6	Arthropoda	*Drosophila melanogaster*	NM_079644.3	NCBI Reference Sequence
*Drosophila melanogaster* NM 079311.3 Rhodopsin 7	Arthropoda	*Drosophila melanogaster*	NM_079311.3	NCBI Reference Sequence
*Apis mellifera* Q17053.1 Rhodopsin	Arthropoda	*Apis mellifera*	Q17053.1	UniProtKB/Swiss-Prot
*Procambarus clarkii* P35356.1 Rhodopsin	Arthropoda	*Procambarus clarkii*	P35356.1	UniProtKB/Swiss-Prot
*Todarodes pacificus* P31356.2 Rhodopsin	Mollusca	*Todarodes pacificus*	P31356.2	UniProtKB/Swiss-Prot
*Mizuhopecten yessoensis* O15973 GQ-coupled SCOP1	Mollusca	*Mizuhopecten yessoensis*	O15973	UniProtKB/Swiss-Prot
*Mizuhopecten yessoensis* O15974 G0-coupled SCOP2	Mollusca	*Mizuhopecten yessoensis*	O15974	UniProtKB/Swiss-Prot
*Octupus vulgaris* AKL61067.1 Rhodopsin	Mollusca	*Octupus vulgaris*	AKL61067.1	GenBank
*Lymnaea stagnalis* evgLocus stringtie AE 32017 Rhodopsin	Mollusca	*Lymnaea stagnalis*	evgLocus_stringtie_AE_32017	Lymnaea.org

#### Protein structural analysis

For protein structure predictions of this *L*. *stagnalis* rhodopsin, AlphaFold Protein Structure Database (RRID:SCR_023662) [36] was used following the standard code in Google Colaboratory. Resulting structures were analyzed in PyMOL (RRID:SCR_000305) for visualizing the 3D structural characteristics of the overall protein folding and architecture, as well as the organization of the secondary structural elements, such as α-helices, β-sheets, and loops, within the protein. Kyte-Doolittle hydrophobicity plots of *Homo sapiens*, *Bos taurus*, and *L*. *stagnalis* rhodopsins were generated using ExPASY ProtScale at a window scale of 15. Intrinsically disordered proteins and regions (IDPs/IDRs) of *B*. *taurus* and *L*. *stagnalis* rhodopsin were predicted using PrDOS (RRID:SCR_021886) [37], with a prediction false positive rate of 5.0%. Additional structures used for comparative analyses were obtained from the protein database: *B*. *taurus* native rhodopsin (1U19) and *Todarodes pacificus* native rhodopsin (2Z73).

### Statistics

One way and two-way analyses of variance and t-tests were carried out in GraphPad Prism 6 (RRID:SCR_002798) to observe any significance in variation and are noted, where applicable.

## Results

### *L*. *stagnalis* retina has structural connections for visual processing within the central nervous system and rhodopsin-positive sensory cells for integration of photo stimuli

**Fig 1:**
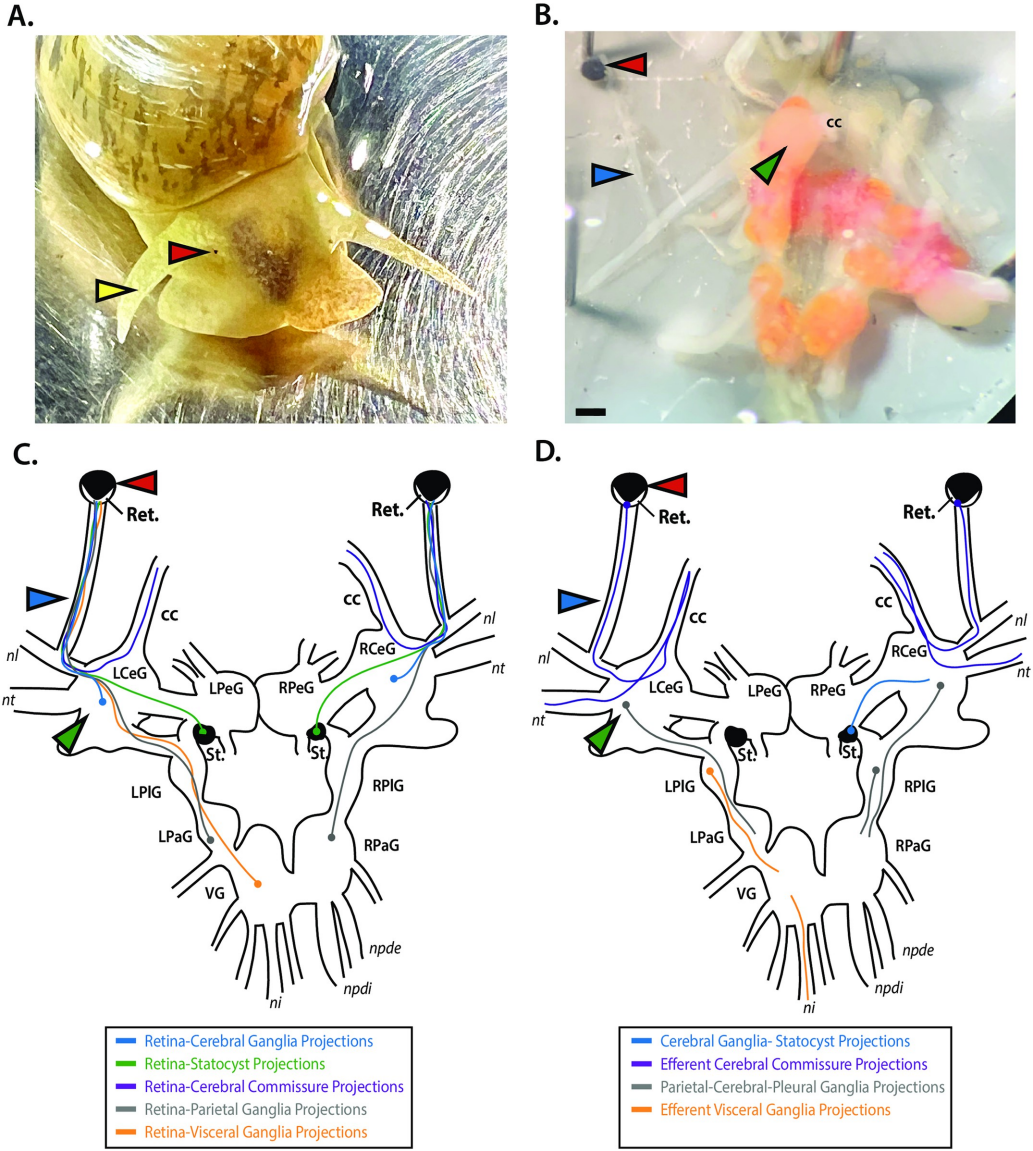
The pathways for sensory information between the *L*. *stagnalis* visual system and the central ring ganglia provides a map to understanding visual processing in gastropod mollusks. **(A)** Depiction of the external anatomy of *L*. *stagnalis*, illustrating anatomical features involved in sensory and visual perception **(B)** Photographed preparation of the internal optic sensory system illustrated by the left eye and left optic nerve connected to the central ring ganglia, via the intermediate n. labialis nerve, facilitating some afferent connections to the cerebral ganglia. Scale bar is indicated (scale bar = 0.5mm). **(C)** Schematic depiction of reported direct afferent projections to the central ring ganglia reported from the retina of both eyes in *L*. *stagnalis* specifically to the cerebral ganglia (blue dashed line), through the cerebral commissure (purple dashed line), to the bilateral statocysts (green dashed line), to the ipsilateral parietal ganglia (grey dashed lines) and to the visceral ganglia (orange dashed line). **(D)** Schematic depiction of select reported efferent (ascending/descending) projections from corresponding retinal afferent terminations in the central ring ganglia. Indicated are the cerebral-statocyst connection (blue dashed line), the crossing cerebral commissure connection to the retina and *n*. *tentacularis* (purple dashed line), from the statocysts to the ipsilateral pedal ganglia (green dashed line), from the parietal ganglia to the ipsilateral cerebral ganglia and the right pleural ganglia (grey dashed lines) and from the visceral ganglia to the left pleural ganglia and through the n. intestinalis to the digestive system (orange dashed line). Dots at the end of each projection indicate terminating points for each projection. Anatomical feature of the tentacles (yellow chevrons), eyes (red chevrons), optic nerves (blue chevrons), and cerebral ganglia (green chevrons) are noted. Ret.- retina, cc- cerebral commissures, LCeG- left cerebral ganglia, LPeG- left pedal ganglia, LPaG- left parietal ganglia, LPlG- left pleural ganglia, RCeG- right cerebral ganglia, RPeG- right pedal ganglia, RPaG- right parietal ganglia, RPlG- right pleural ganglia, VG- visceral ganglia, St.- statocyst, nl- *n*. *labialis*, nt- n. *tentacularis*, ni- *n*. *intestinalis*, npdi- *n*. *pallialis dexter internus*, npdi- *n*. *pallialis dexter externus*.

To establish an anatomical foundation for the use of *L*. *stagnalis* as a model of vision-based locomotory behaviour, we first characterized the structural anatomy and connectivity of the *L*. *stagnalis* visual system. The two pigmented eyes (red chevrons) thought to form the basis of binocular vision in *L*. *stagnalis* sit adjacent to tentacles (yellow chevrons) extending from the head (Fig 1A). Through preparations of the *L*. *stagnalis* central ring ganglia with attached optic fibers (Fig 1B), branching of the optic nerve (blue chevron) with a larger, peripheral nerve (*n*. *tentacularis*) is observed, as well as the connections to the cerebral ganglia (green chevron) (Fig 1B) [14], allowing retinal photoreceptor axons to form afferent innervation of the central ganglia ring and/or statocyst. Also, some central neuron axons produce efferent projections to the retina. Among these central neurons, some projections innervate both eyes, providing the cellular basis for binocular vision [13]. To better understand the numerous retinal projections to/from the CNS, previous studies were examined. Through the optic nerve, several first-order projections from the retina reside within the central ring ganglia (Fig 1C), where the majority end at the cerebral ganglia (retina-cerebral ganglia projections; blue) [13], at the statocyst via retina-statocyst projections (green), at the visceral ganglia via retina-visceral ganglia projections (orange), or at the parietal ganglia via retina-parietal ganglia projections (grey). Binocular information is also communicated contralaterally via the retina-cerebral commissure pathway (purple) [38]. Second-order projections from first-order retinal sites (Fig 1D) proceed from the cerebral ganglia to the statocyst via cerebral ganglia-statocyst projections (blue). Interestingly, contralateral retina-cerebral commissure projections (purple) not only terminate in the contralateral retina, but also form efferent projections to skin via the *n*. *tentacularis* nerve. While inter-ganglia projections from the parietal ganglia to the pedal and pleural ganglion exist via parietal-cerebral-pleural ganglia projections (grey), and additional secondary projections from the visceral ganglia traverse via the *n*. *intestinalis* nerve to form efferent visceral ganglia projections (orange), the termination point(s) of these later projections are unknown.

**Fig 2:**
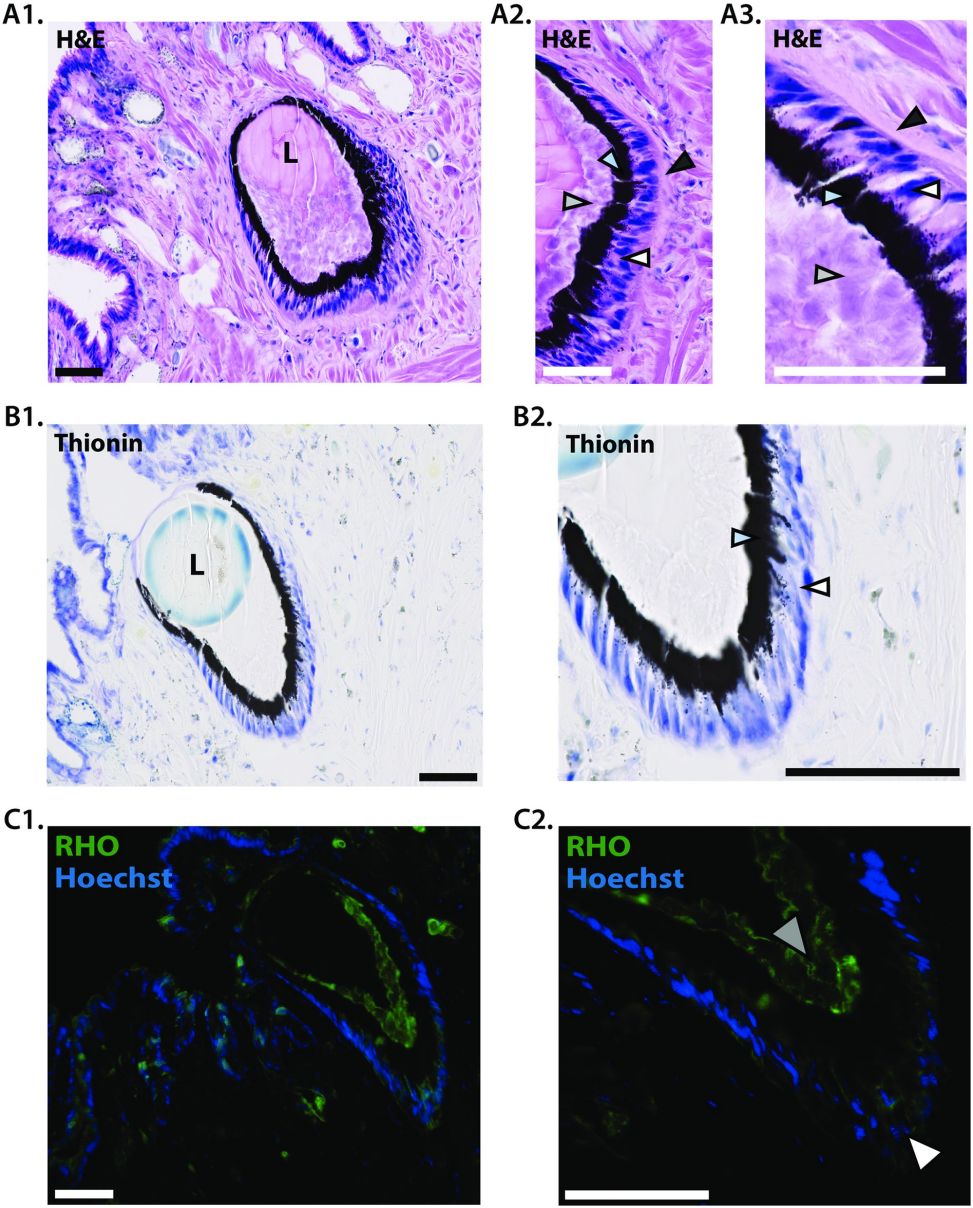
Morphological characterization of the *L*. *stagnalis* eye illustrates the presence of key anatomical features involved in phototransduction and visual perception. **(A)** Hematoxylin and eosin-stained sections of the *L*. *stagnalis* eye depict the general histology and structure of the dermal and ocular tissues. **(B)** Thionin stained section of the *L*. *stagnalis* eye depicts the dense nuclei staining in the somatic layer of retina. **(C)** Immunofluorescence staining of the *L*. *stagnalis* eye illustrating the general distribution of the cell nuclei and rhodopsin positive cells in the photopigment and somatic retinal layers of the eye and the peripheral dermal tissue. Hoechst-2293 positive nuclei are indicated in blue, and rhodopsin positive cells are stained with Anti-Octopus rhodopsin in green. Photopigment positive dermal photoreceptor cells (yellow chevrons), rhabdomeric membranes (grey chevrons) and somatic photopigment cells (white chevrons) in the retina are noted, and the lens is denoted ‘L’. Sections—7μm, Scale bars—50 μm.

To better understand the sensory components driving potential light-sensitive responses given the mapped neural pathways for visual information, we sought to obtain histological and anatomical information on the *L*. *stagnalis* ocular tissues. Hematoxylin/eosin (H&E) staining of *L*. *stagnalis* eye slice preparations reveal a circular cup-like eye structure, with multi-layered retina (Fig 2-A1). Posterior to the large lens located within a vitreous body, four layers of the *L*. *stagnalis* retina are present (Fig 2-A1), with pigmented granules positioned between the two retinal layers (blue chevron; Fig 2-A2). Retinal layers are organized in a laminar fashion from the lens (Fig 2-A3); an epithelial layer (light grey chevron), a pigment layer (blue chevron), a nucleated somatic layer (white chevron) and a neuronal layer (dark grey chevron). To complement these findings, we conducted thionin staining and identified retinal layers containing cell bodies, denoted by dark blue staining (Fig 2-B1) between retinal pigment and neuronal layers (Fig 2-B2). Notable, is the microvilli layer that is devoid cell nuclei. The cell nuclei of the somatic layer found between the pigment layer and neuronal layer are also stained in the H&E preparations (Fig 2- A2, A3), suggesting that the neuronal layer houses most, if not all, of the cell nuclei within the retinal layer. Of note is the absence of retinal tissues at single point within the eye cup, indicating the location of the putative cornea [39]. To characterize which layers of the retina, mediate the initiation of phototransduction in the eye, we identified rhodopsin-positive cells using a commercially available anti-rhodopsin antibody identifying rhodopsin in *L*. *stagnalis* [23]. We identified that the rhabdomeric membranes of the *L*. *stagnalis* eye house -positive retinal cells in several layers of the retina (Fig 2-C1), and this staining appears to wrap around the eye cup structure with Hoescht-2293 staining overlapped with rhodopsin-positive signatures (Fig 2-C2), consistent with previous studies [23, 40].

**Fig 3:**
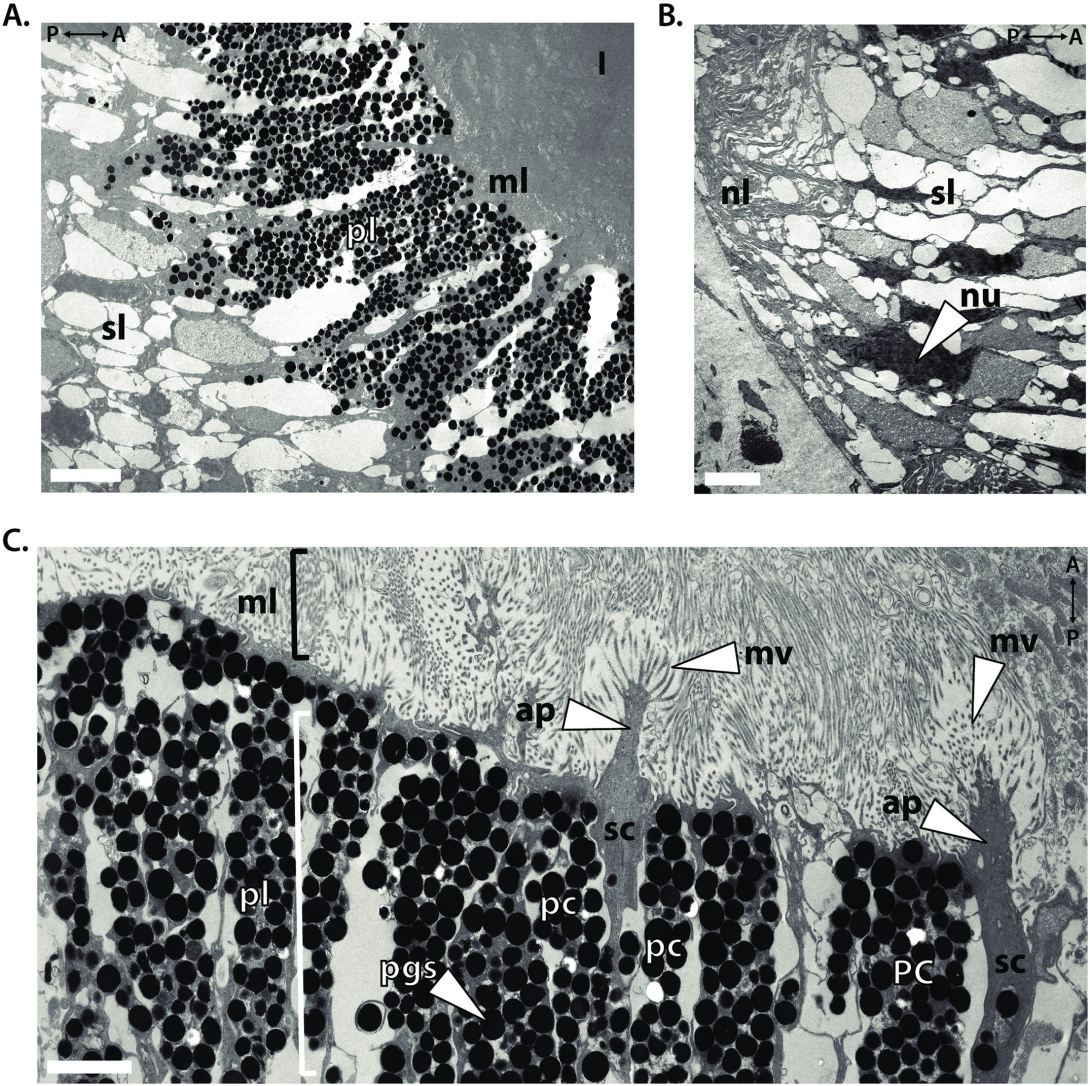
Electron microscopy of *L*. *stagnalis* retinal ultrastructure reveals detailed organization of the layered retina and distinct retinal subtypes. Electron microscopic images of **(A)** longitudinal cross sections of the *L*. *stagnalis* retina show, from anterior to posterior, lens (*l*), microvillar layer (*ml*), pigment layer (*pl*), the somatic layer (*sl*), **(B)** and the neural layer (*nl*). (C) Higher magnification electron micrographs of the photoreceptor sensory cell (*sc*) and pigment cell (*pg*) distribution within the microvilli and pigment layers of the retina are shown, with the apical projections (*ap*) and microvilli (mv) indicated. Pigment granules (*pgs*) of the pigment layer are labelled. Directionality from the anterior (A) to posterior (P) portions of the eye are indicated. Sections—70 nm, Scale bars: **A** = 5 μm, **B** = 5 μm, **C** = 1 μm.

To gain greater insights in *L*. *stagnalis* retina ultrastructure and speculate on the morphology of the photo-sensitive rhabdom, we performed electron microscopy on 70 nm thick retinal tissue slices. Here, we found that the pigment layer, measuring approximately 10–25 μm, is abundant in electron dense pigment granules (Fig 3A). The pigment layer is positioned posterior to the microvilli layer (*ml*), which measures 2–5 μm and lies adjacent to the lens (*l*). The microvilli layer is positioned as the proximal most retinal layer to the cornea (not shown) making it the most anterior of the retinal layers (Fig 3A). The somatic layer (*sl*) lies posterior to the pigment layer and houses electron dense nuclei (nu). The somatic layer lies adjacent to the neural layer, which measures 5–10 μm and is the most distal retinal layer from the lens and exhibits long (Fig 3B). Higher magnification electron microscopic images revealed single apical projections (*ap*) from photoreceptor sensory cells (*sc*) and the extending sensory cell microvilli (*scmv*) of the rhabdom, which together measure 2.0–2.5 μm each within the microvilli layer (Fig 3C). Several additional microvilli projections (*mv*), presumably from the pigment cells, are present and lay adjacent to the rhabdoms of the sensory cells (Fig 3C). At higher magnifications, the size of the pigment granules become more visible, measuring 250–500 μm. Pigment granules appear to be more densely packed in the pigment cells of the pigment layer, with few granules being present in the region of the photoreceptor sensory cells that lies within the pigment layer (Fig 3C).

### Neurobehavioral testing through DeepLabCut reveals that most snails exhibit a positive phototaxis response to intense focal light

**Fig 4:**
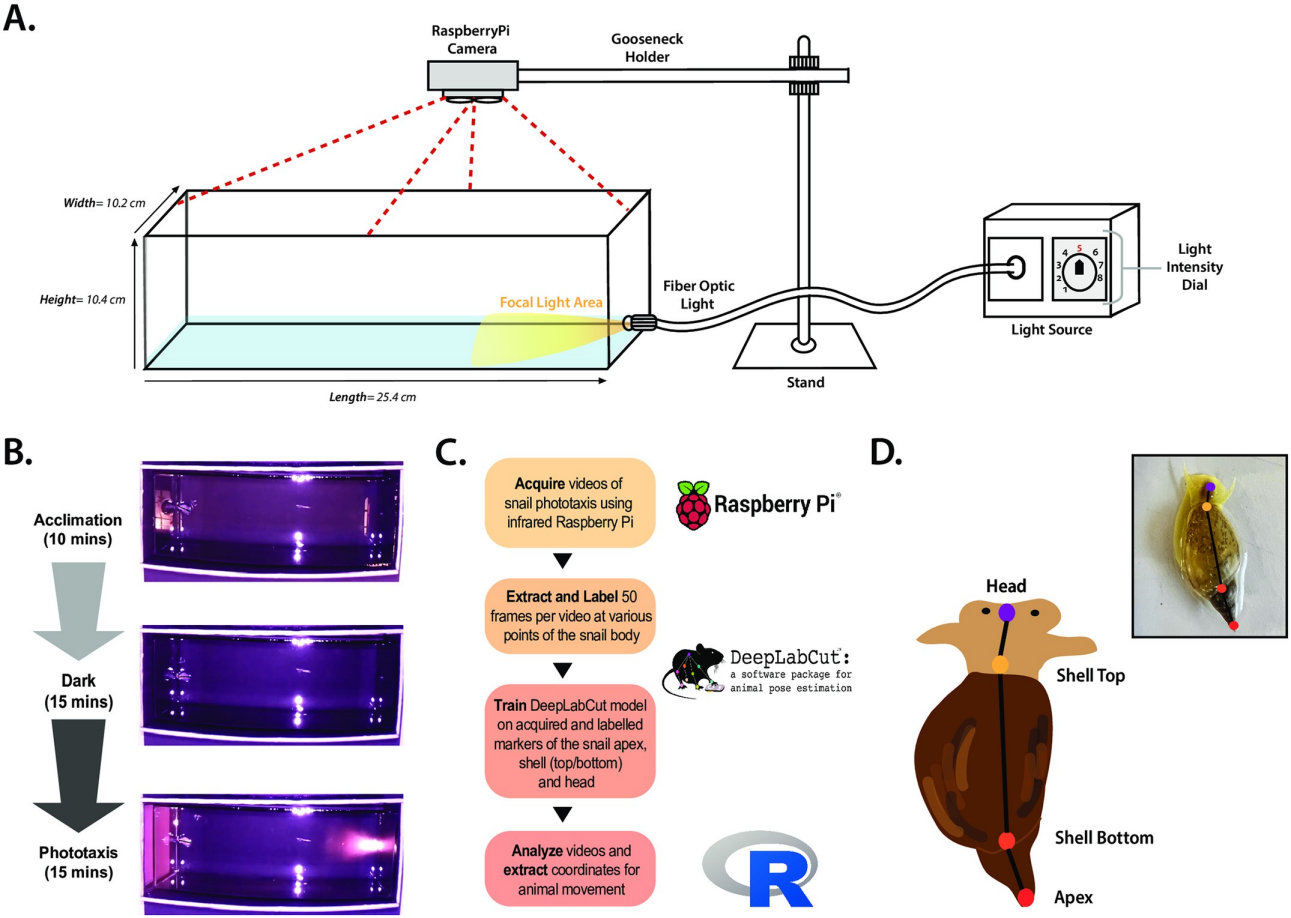
Development of a neurobehavioral protocol using DeepLabCut to assess snail phototaxis response *in vivo*. **(A)** Diagram of the experimental phototaxis arena where snails are tracked by an overhead infrared RaspberryPi camera while performing locomotion. **(B)** Pictorial representation of phototaxis testing protocol example frames from each of the three phases—*acclimation*, *dark*, and *focal light-* and the recording time for each phase. Noted is the consistent positioning of the snail on the opposite end of the focal light source at the start of each phase. **(C)** Schematic drawing and representative image collected from RaspberryPi recordings. Snail labelling of the head region (purple dot), top of the shell (yellow dot), bottom of the shell (orange dot) and shell apex (red dot) are done in the DeepLabCut GUI [26]. **(D)** General pipeline for DeepLabCut analysis of snail phototaxis behavior. Videos of snail phototaxis (20 fps) were acquired on RaspberryPi computers with recording infrared cameras and extracted from the computer for analysis through DeepLabCut. 50 frames from each video were extracted and critical body parts were labelled in the DeepLabCut GUI. Once labelling was completed, labelled frames were used to train the DeepLabCut machine learning model neural network on the placement of the labelled body parts, with the apex being the most consistent label throughout all videos, to learn the animals’ movement during phototaxis testing. Videos were then analyzed and the coordinates for each labelled body part were extracted to determine various parameters of animal movement.

**Fig 5:**
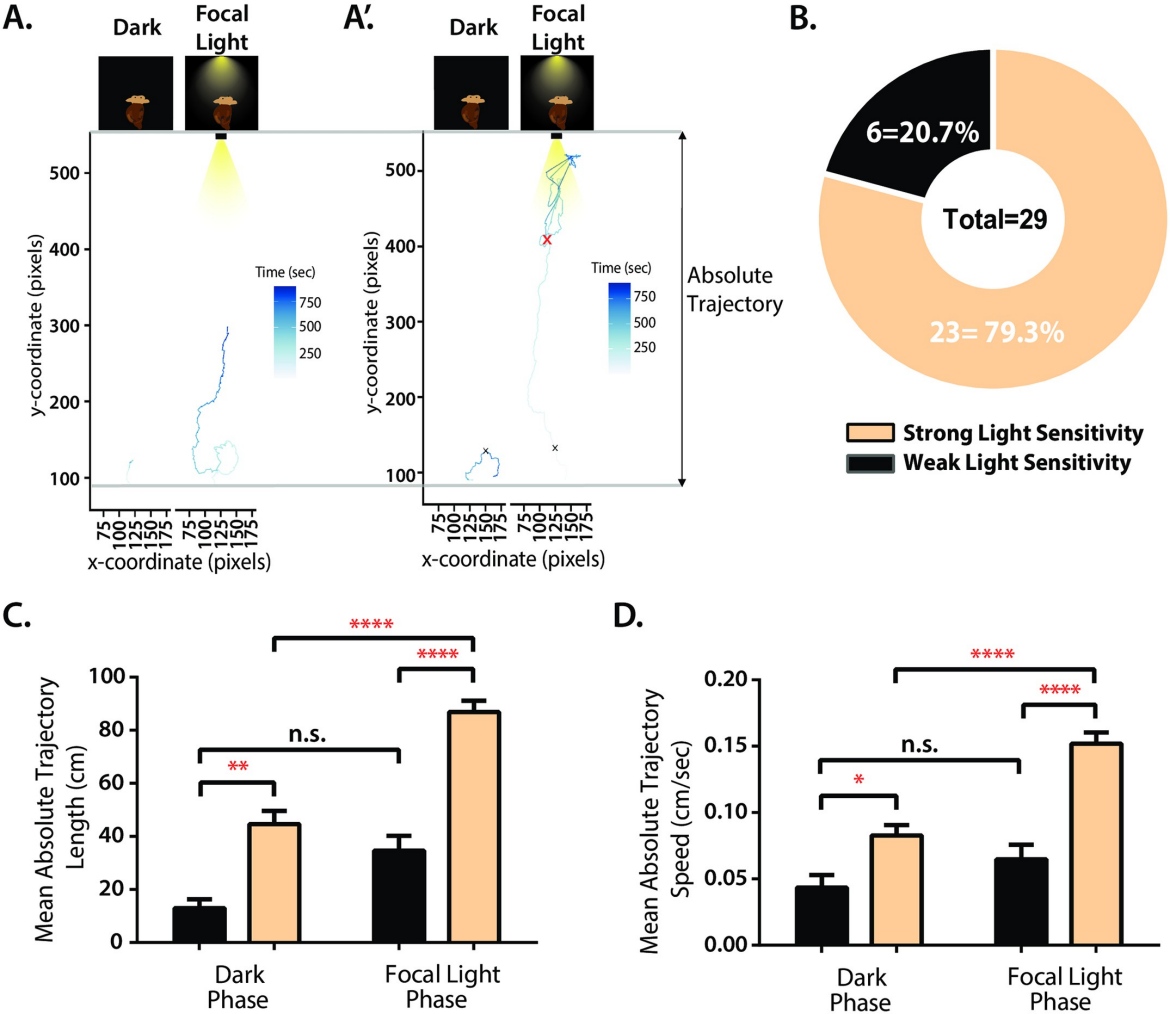
Assessing phototaxis abilities *in vivo* reveals that most animals exhibit patterns of positive phototaxis. Representative travel trajectory plots of snails who **(A)** exhibit weak light sensitive behaviors and **(A’)** strong light sensitive behaviors, where the boundaries by which absolute trajectory length was calculated are noted. **(B)** Chart depicting the total number/ percentage of animals within the testing cohort (n = 29) who reached the focal light area during the focal light phase, exhibiting strong light sensitivity (n = 23, 79.3%) and those who did not enter the focal light area during the focal light phase, therefore exhibiting weak light sensitivity phototaxis (n = 6, 20.7%). Comparative plots of **(C)** mean absolute trajectory length travelled and **(D)** mean absolute trajectory speed during the dark and focal light phases of testing for strong light sensitive (n = 23) and weak light sensitive (n = 6) animals. For panels (C, D), unpaired two-way ANOVA and Tukey’s multiple comparisons tests were performed and statistical significance, if any, were reported. (* = p < 0.05; ** = p < 0.01; *** = p < 0.005, **** = p < 0.001; n.s. = not significant; two-way ANOVA with Tukey’s multiple comparison). +/-SEM for all groups are noted.

To determine whether the *L*. *stagnalis* visual system can generate a phototactic response, we constructed a neurobehavioral test to assess snail locomotory movement in response to intense focal light (Fig 4A and 4B). To do this, we assessed the *L*. *stagnalis* ‘gliding’ movement, where the animal could only move within XY planes of the arena, in a shallow surface toward a focal light presentation at the opposite end of the rectangular arena (see S1 File). Two clear patterns of photosensitive movement appeared within our testing cohort (n = 29), 20.7% (6/29) demonstrated weak responsivity to the applied source, whereas 79.3% (23/26) exhibited strong position response to the applied light source (Fig 5A and A’, respectively, see Fig 5B). All light-sensitive animals were observed to locomote during both dark and focal light phases, with trajectory lengths significantly higher for phototactic snails during the focal light phase (86.85 ± 4.235) than during the dark phase (44.58 ± 5.081) as shown in Fig 5C (two-way ANOVA with multiple comparisons p<0.0001). Similarly, snails exhibiting weak phototaxis demonstrated a significant increase in absolute trajectory lengths when exposed to light (34.63 ± 5.562) compared to the dark phase (12.95 ± 3.318; two-way ANOVA with multiple comparisons p = 0.0068), suggesting that the nature of the phototactic response is similar in both groups, differing only in their degree of locomotion. Snails that did not move during both the dark and focal light phases were not included in the study. Phototactic snails in this cohort have significantly higher absolute trajectory lengths during the focal light phase compared to weakly light-sensitive snails (two-way ANOVA with multiple comparisons p< 0.0001), though this difference was also observed during the dark phase, demonstrating the tendency for snails to explore the arena even in the absence of strong focal light.

In addition to phototaxis, aquatic animals also sense and react to the presence of light by exhibiting photokinesis, or increased speed induced by the presence of light [41, 42]. Thus, to determine whether phototactic snails exhibit photokinesis via an increase in speed of movement, we determined the mean speed of each animal during total trajectory locomotion in both dark and focal light phases. Phototactic animals display a significant two-fold increase in speed in the presence of focal light (0.10 ± 0.0060) compared to in the dark phase (0.053 ± 0.0063; two-way ANOVA multiple comparisons p<0.0001) (Fig 5D). Weakly light sensitive snails demonstrate lower mean speeds than phototactic snails during both the dark (0.015 ± 0.0036; two-way ANOVA multiple comparisons) and focal light (0.039 ± 0.0066; two-way ANOVA multiple comparisons) phases. Despite their lower overall speeds in both conditions, weakly light-sensitive snails also demonstrate a roughly 2.5-fold increase in mean speed in the presence of focal light than in the dark phase. Together, these data suggest that phototactic animals also demonstrate stronger photokinetic responses than weakly light-sensitive animals.

### Phylogenetic and protein signature analysis reveals how the unique *L*. *stagnalis* rhodopsin may be key to providing insights into mollusk phototaxis and light-sensitivity modalities

**Fig 6:**
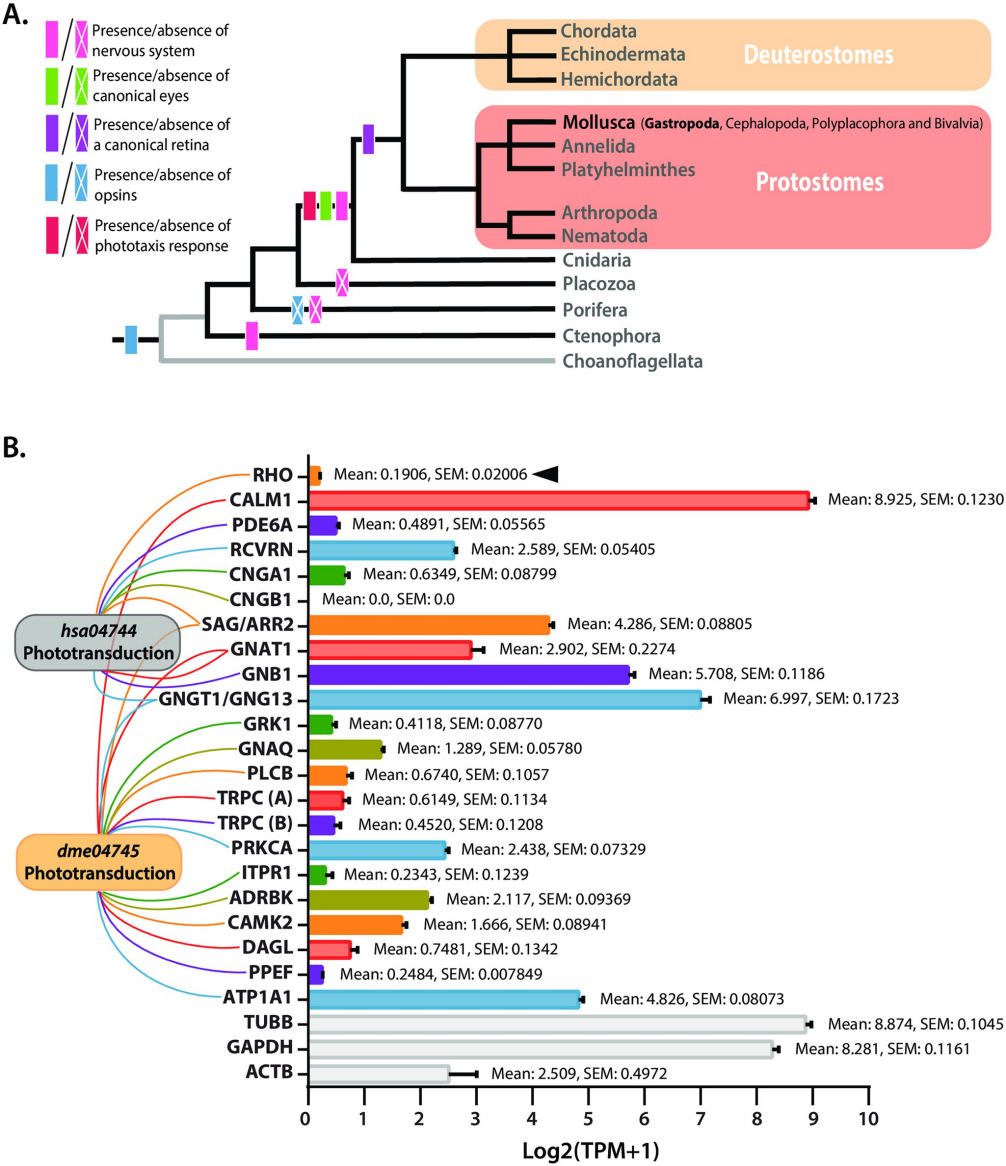
The conservation patterns in the phototransduction pathway provides insights on mollusk light sensing modalities. **(A)** Phenotypic patterns of critical components of visual system structure and function across eukaryotic animals are depicted in this phylogenetic tree rooted in choanoflagellates as the most divergent group of eukaryotes with photo-sensitive properties. While the evolution of photo opsins and the phototaxis behavior appears to predate the evolution of the canonical nervous system in Ctenophora, Cnidaria, and Bilateria, the presence of sensory eye organ and an organized retina appear to be exclusively found in Bilateria. Evident is the evolution of canonical retina and nervous systems in Mollusca and other protostomes, as well as the presence of phototaxis behaviors in bilaterians. Of note, this tree supports the hypothesis that ctenophores are the most early diverging animals at the base of Metazoans, not poriferans (i.e., sponges). **(B)** Average TPM expression level analysis of the *L*. *stagnalis* vertebrate and invertebrate phototransduction pathway homologs, mined from the *L*. *stagnalis* CNS (i.e., central ring ganglia) transcriptome [27] reveals reduced expression of both invertebrate and vertebrate phototransduction genes in the CNS of the snail. Reference genes *glyceraldehyde-3-*phosphate dehydrogenase *(GAPDH)*, beta-actin *(ACTb)*, and beta-tubulin *(TUBB)* were included in this study. Mean and +/-SEM for all mined genes are noted.

Having established that *L*. *stagnalis* display robust phototactic and photokinetic responses, we next sought to identify which critical phototransduction molecules are conserved and thus characterize the molecular machinery available for visual system function in this organism. To determine whether mollusks bear the evolutionarily conserved features critical to phototaxis we constructed a phenotypic phylogenetic tree based on visual system components and assessed the presence or absence of a phototactic response in each clade (Fig 6A). This tree, rooted in choanoflagellates as the most divergent group of eukaryotes and the protist sister group to animals, shows choanoflagellates and animals as exhibiting photosensitivity, and choanoflagellates and early diverging organisms (Ctenophora, Porifera, and Placozoa) lacking canonical phototaxis responses [43]. Importantly, while the evolution of photo opsins, eyes and phototaxis behavior appears to predate the evolution of the canonical nervous system in Mollusca and other bilaterians as well as the early diverging Ctenophora and Cnidaria, the presence of sensory eye organ and an organized retina appear to be exclusively found in bilaterian deuterostomes and protostomes, and some cnidarians (i.e., Cubozoa, Scyphozoa and Hydrozoa), where the later possess both complex eye structures [44, 45] and light-guided visual responses [46]. Like other bilaterian phyla, such as Arthropoda [47, 48], there is broad diversification of eye types in Mollusca where some mollusks bear simpler eye structures (i.e., chitons) [49–51], and some that do not bear eyes at all (i.e., Aplacophora, Monoplacophora and Scaphopoda), though the majority of identified species bear complex and/or specialized eyes [52]. This may indicate that the unique diversification of eyes structures within Mollusca are species-specific and may have multiple origins, thereby strengthening the need to characterize eye structures and light-guided visual responses in animals throughout this phylum. As well, the evolution of canonical eyes and a retina proceeds the onset of the nervous system in bilaterians, which may indicate that the evolution of visually guided behaviors to light (i.e., phototaxis) in mollusks requires a synergy between complex CNS and eye evolution.

Mollusks, including *L*. *stagnalis*, appear to have critical phenotypic components of vision, and given that several ion channels, including some that may be involved in snail phototransduction, are conserved in *L*. *stagnalis* [27], we next screened for the absence/presence of select molecular identities required for canonical vertebrate and invertebrate phototransduction. First, using the human phototransduction KEGG pathway proteins as query for our search, we identified homologs of the following genes within the translated *L*. *stagnalis* transcriptome that are known to be critical to vertebrate phototransduction: rhodopsin (RHO), calmodulin 1 (CALM1), phosphodiesterase 6A (PDE6A), recoverin (RCVRN), cyclic nucleotide-gated channel alpha 1 (CNGA1), cyclic nucleotide gated channel beta 1 (CNGB1), S- arrestin (SAG), G protein subunit alpha transducin 1 (GNAT1), G protein subunit beta transducin 1 (GNB1), G protein subunit gamma transducin 1 (GNGT1), and rhodopsin kinase (GRK1), but failed to identify a putative homolog for Retinal Outer Segment Membrane Protein 1 (ROM1) (Table 1). As well, given mollusks phylogenetic placement and the molecular differences between vertebrate and invertebrate phototransduction signalling processes, we also identified select homologs within the translated *L*. *stagnalis* transcriptome using the *Drosophila melanogaster* phototransduction KEGG pathway genes; rhodopsin (RHO), guanine nucleotide-binding protein G(q) subunit alpha (GNAQ), guanine nucleotide-binding protein G(I)/G(S)/G(O) subunit gamma-13 (GNG13), phosphatidylinositol phospholipase C, beta (PLCB), classical protein kinase C alpha type (PRKCA), calmodulin (CALM), arrestin-2 (ARR2), inositol 1,4,5-triphosphate receptor type 1 (ITPR1), beta-adrenergic-receptor kinase (GRK), calcium/calmodulin-dependent protein kinase (CaM kinase) II (CAMK2), sn1-specific diacylglycerol lipase (DAGL), and serine/threonine-protein phosphatase with EF-hands (PPEF, PPP7C). Our search into transient receptor potential cation channels (TRPC), critical to the invertebrate phototransduction pathway, yielded two putative TRPC-like channels, dubbed TRPCA and TRPCB. Queries for SAG and ARR2 yielded the same result, which we dubbed SAG/ARR2, as did those for GNGT1 and GNG13, thusly dubbed GNGT1/GNG13. Notably, we failed to identify a putative homolog for the inactivation no afterpotential D protein (INAD). We next assessed transcript per million (TPM) expression data for all the mined phototransduction pathway homologs of interest, indicating that, apart from the CALM1 homolog, most phototransduction-related homologs were not enriched, but were present in the central ring ganglia when compared to reference proteins beta-actin (Act_b_), Glyceraldehyde 3-phosphate dehydrogenase (GAPDH) and beta-tubulin (TUBB), which are all abundantly expressed in the CNS [28]. Notably, the identified *L*. *stagnalis* RHO homolog (*Log2(TPM+1*); 0.1906 ± 0.021) has a significantly lower copy number in CNS tissue when compared to the reference proteins (one way ANOVA Dunnett’s multiple comparisons; RHO vs Act_b_ p<0.05; RHO vs GAPDH p<0.0001; RHO vs TUBB p<0.0001)(Fig 6B), but was still present in the CNS despite the later not being the primary visual system organ.

**Fig 7:**
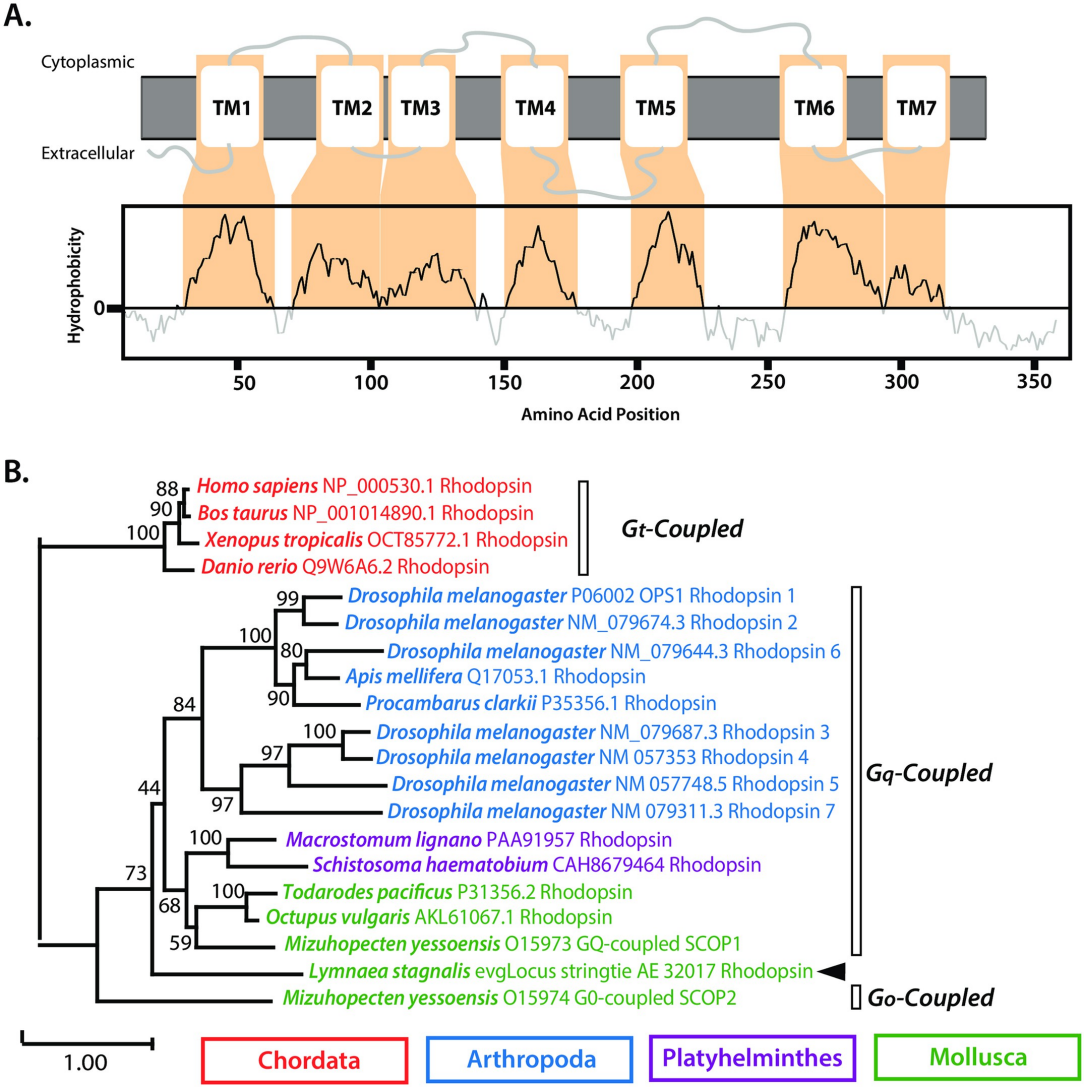
*In silico* characterization of *L*. *stagnalis* RHO reveals a unique phylogenetic placement despite its canonical GPCR rhodopsin structure. **(A)** Kyte-Dootlittle plot depicting transmembrane (>1) and cytoplasmic/extracellular (<1) spanning regions of *L*. *stagnalis* rhodopsin (RHO) reveals seven hydrophobic regions corresponding to seven transmembrane spanning alpha-helices. **(B)** Phylogenetic tree of various vertebrate and invertebrate rhodopsin proteins from model organisms critical to vision research, inferred by IQ tree using the LG+F+I+G4 model. Node support values resulting from 1,000 ultra-fast bootstrap replicates are indicated, and the scale bar indicates the number of amino acid substitutions per site. Phylogenetic subgroups of rhodopsins engaged in G-protein coupled mechanisms through Gt-, Gq- and Go-protein interactions are noted, revealing that *L*. *stagnalis* rhodopsin does not cluster with the canonical vertebrate and invertebrate rhodopsin and may function through unique G-coupled mechanisms.

Due to the deep conservation of opsins in eukaryotes and the role of rhodopsin at the critical initiator of phototransduction in later diverging animals [53], we sought to conduct further phylogenetic analyses of the predicted *L*. *stagnalis* rhodopsin homolog (RHO) in the *L*. *stagnalis* CNS, which houses photo-sensitive cells [54]. Analysis of *L*. *stagnalis* RHO topology through Kyte-Doolittle hydrophobicity analysis found seven predicted transmembrane spanning hydrophobic domains (Fig 7A). Given that invertebrate rhodopsins tend to couple with Gq-proteins as opposed to vertebrate Gt- proteins, we sought to predict, based on phylogenetic placement of *L*. *stagnalis* RHO, the identity of the G-protein it may couple with. To predict whether *L*. *stagnalis* RHO is more closely related to vertebrate or invertebrate rhodopsins, and thus may engage in similar coupling mechanisms, we constructed a maximum likelihood phylogenetic tree of rhodopsin proteins (Table 2) from animals commonly used as models in vision sciences and/or phototaxis (i.e., vertebrates, arthropods, and mollusks) (Fig 7B). Of note, while not commonly used as a model for invertebrate vision sciences, select platyhelminth animals who have robust transcriptomes available were included as they have complex visual system comprised of both photosensitive rhabdomeric and ciliary membranes with pigmented cells [51], and may serve as an intermediate organism for the evolution of the visual system between lophotrochozoans and ecdysozoans, thusly providing insight on the phylogenetic placement of *L*. *stagnalis* RHO. Surprisingly, *L*. *stagnalis* RHO did not cluster with other molluscan rhodopsin homologs that are Gq-coupled or with Go-coupled *M*. *yessoensis* SCOP2, instead forming its own branch 73% of the time.

### Structural analysis into *L*. *stagnalis* rhodopsin alludes to conserved capacity for photoreceptor-mediated light-sensitive activity

**Fig 8:**
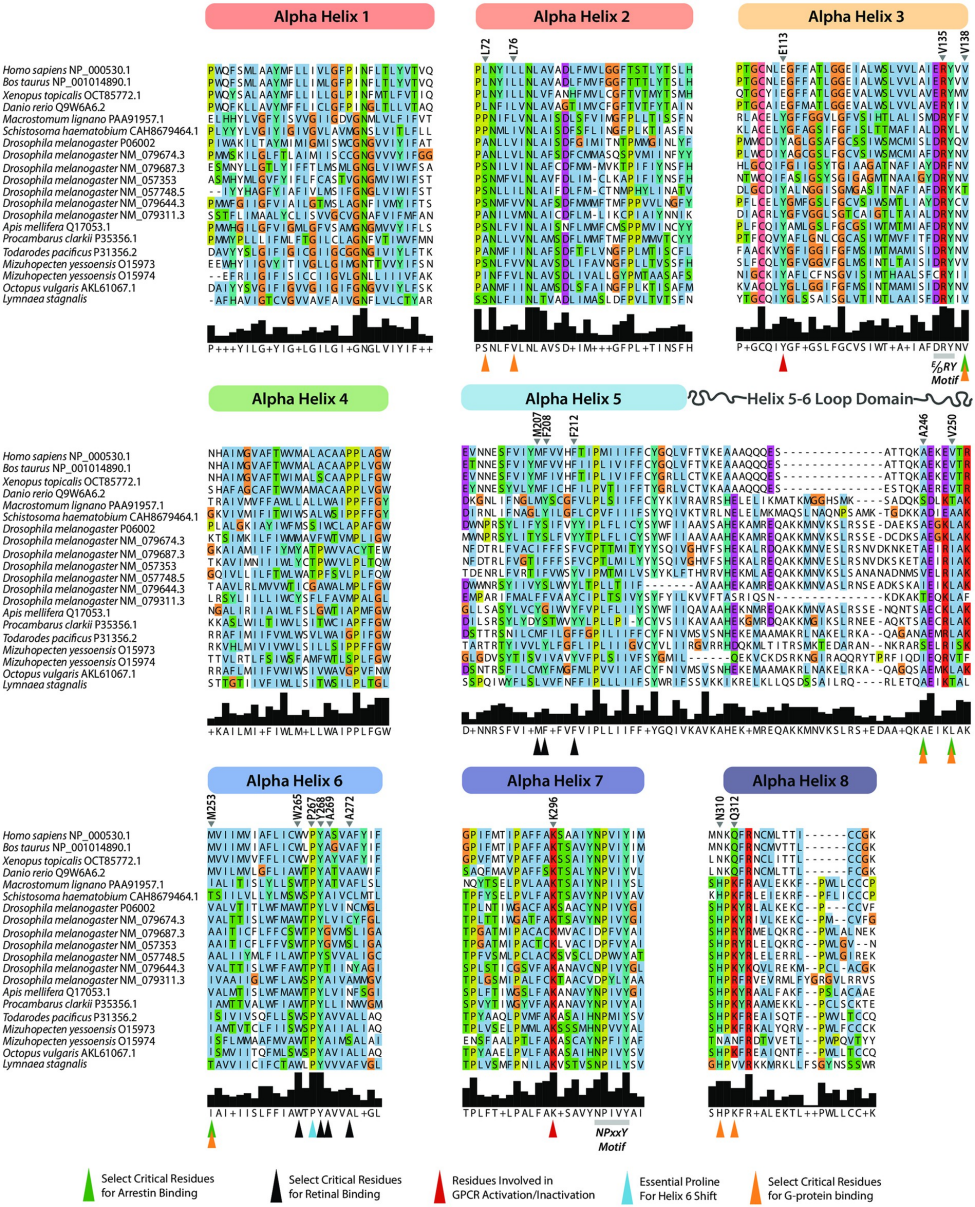
Evolutionary conservation of critical amino acid residues to rhodopsin’s light-sensitive function in vertebrate rhodopsins are present in gastropod mollusks. Protein sequence muscle alignment of rhodopsin alpha-helices, identified through PROMALS3D, reveals deep conservation of rhodopsin proteins among representative animals in animal phyla commonly referenced in vision sciences. Amino acids residues involved in arrestin binding (green chevrons), comprising the major retinal ligand binding pocket of rhodopsin are shown (black chevrons), rhodopsin GPCR activation/inactivation (red chevrons) and G-protein binding (orange chevrons), and additional amino acids that cooperate with those involved in lining the ligand binding pocket but are preferentially involved in photoactivation of 11-cis-retinal are indicated (blue chevrons) are indicated. Of note are the conserved NPxxY and D(E)RY motifs, which are involved in rhodopsin’s conformational change in response to retinal photoisomerization and the ability of rhodopsin to reach the active conformation, respectively.

Given that our rhodopsin phylogenetic assessment suggested that a putative *L*. *stagnalis* RHO may group outside of the Gq- and Gt-coupled rhodopsins, we next sought to characterize the critical protein sequence signatures of *L*. *stagnalis* rhodopsin and assess conservation between the putative *L*. *stagnalis* RHO and the other vertebrate and invertebrate rhodopsins included in our study. A MUSCLE protein sequence alignment of all aligned rhodopsin alpha-helices and the helix 5–6 loop domain was constructed of rhodopsin proteins from the representative animals included in the phylogenetic analysis (Fig 8). The TM 5–6 loop domain is of particular interest given the conformational changes that occur to accommodate G-protein binding after photoactivation, namely the cytoplasmic end of transmembrane helix 6 (TM6) straightens and shifts away from the molecule’s center and transmembrane helix 5 (TM5) experiences a minor repositioning and rotation [55, 56]. Notably, the alpha-helix structure of *L*. *stagnalis* RHO appeared to be well conserved, noting seven predicted transmembrane bound alpha-helices (TM1-TM7) and one transverse alpha-helix (H8) that were strongly aligned with the other sequences, with consensus sequence noted. Like other invertebrate rhodopsins, *L*. *stagnalis* RHO has a longer TM 5–6 loop (~10 amino acids) than the vertebrate rhodopsins. Noteworthy is the conservation of the NPxxY motif in TM5 and the D(E)RY motif in TM3 in both vertebrate and invertebrate rhodopsins. These motifs are both involved in conformational transformations of rhodopsin that are required for receptor activation and transformation to metarhodopsin [57]. As well, the essential proline (P267) located in TH6 (Fig 8; blue chevron) that is required for a proline hinge to create an outward shift of TM6 upon photoactivation [56] is deeply conserved across surveyed species.

Next, guided by literature, we assessed whether critical residues required for rhodopsin activation, E113 and K296 (Fig 8; red chevrons), were conserved in *L*. *stagnalis* RHO. Importantly, these residues in mammals are involved in inactivation/activation of the channel, where mutation to either of these amino acids produces constitutive activation of the receptor [58]. Despite not bearing the mammalian E113 residue, *L*. *stagnalis* RHO bore the conserved tyrosine (Y) residue found exclusively in invertebrates at this position, while K296 appeared to be strongly conserved throughout the invertebrate phyla. Like the rhodopsin activation residues, those hydrophobic residues involved in retinal binding (black chevrons) also appeared to be well conserved amongst invertebrates, suggesting that *L*. *stagnalis* RHO has the capacity to bind retinal in a similar manner to other animals. Importantly, these hydrophobic residues predate the evolution of metazoans [59], though some invertebrate animals included in our analysis do not appear to have the hydrophobic residues required for retinal binding, namely in TM5 (mammalian M207, F208 and F212), which are involved in, but not solely responsible for, stabilizing the retinal binding pocket and the conformational change in rhodopsin upon activation [60].

We also investigated whether the residues required for rhodopsin-arrestin complexing in mammals, A246, V250 and M253 [56], were present in *L*. *stagnalis* and found that A246 is present in *L*. *stagnalis*, but V250 and M253 are absent (green chevrons). Finally, to determine whether residues involved in mammalian G-protein binding (orange chevrons) are present in *L*. *stagnalis* RHO, we surveyed whether these residues spanning the transmembrane helices are conserved in mollusks. We found that several residues involved in G-protein binding (orange chevrons) also facilitate binding to arrestin (green chevrons) in vertebrates [56, 61], like V138, A246, V250 and M253, while N310 and Q312 in H8 are specific to G-protein binding and are not well conserved in select mollusk rhodopsins (*M*. *yessoensis* O15974 and *L*. *stagnalis* RHO). As well, some invertebrate residues that evolved prior to the evolution of vertebrates and may be specific to Gq-type binding are also absent in *L*. *stagnalis* RHO, such as V266 in TH6 and Q312 in TH8.

**Fig 9:**
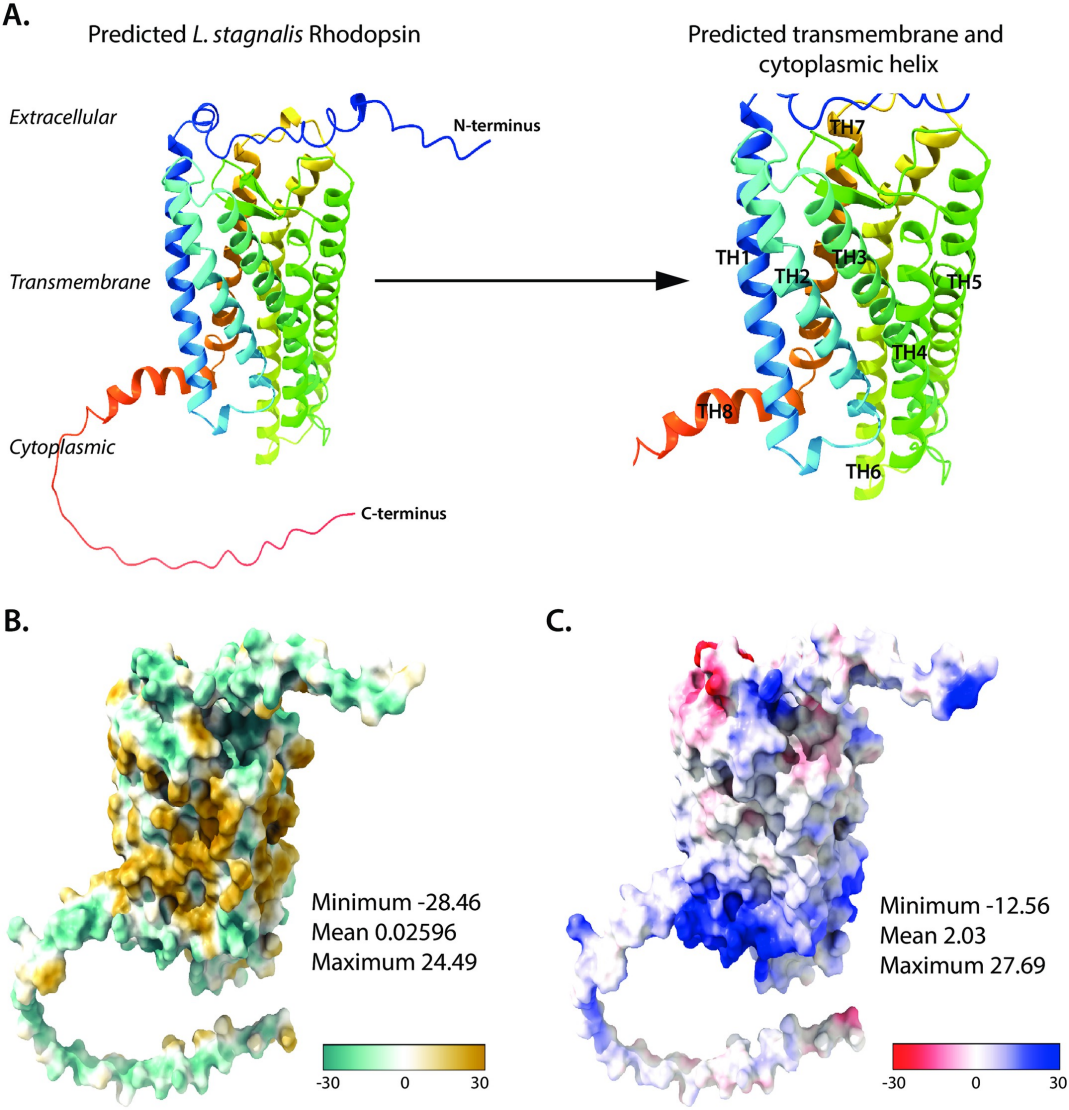
AlphaFold2 predicted structure of *L*. *stagnalis* rhodopsin. **(A)** Multicolored AlphaFold2-predicted secondary structure of the putative *L*. *stagnalis* rhodopsin with the N- and C- termini of the structure located within the extracellular and cytoplasmic regions, respectively, indicated. Also depicted on this structure are the predicted seven transmembrane and one cytoplasmic helix (TH1-TH8) noted. The *L*. *stagnalis* structure surface was colored to indicate **(B)** Electrostatic surface potential and **(C)** hydrophobicity, with the minimum, mean, and maximum scores for these calculations and scales pertaining to these parameters are noted.

To determine whether these non-conserved amino acid sequences of putative *L*. *stagnalis* RHO were associated with differences in protein structure, we first modelled the putative *L*. *stagnalis* RHO using AlphaFold2 to predict *in silico* its 3D structure from its predicted amino acid sequence (Fig 9A). Consistent with our protein alignment, 3D putative *L*. *stagnalis* RHO has eight predicted α-helix structures; seven transmembrane spanning helices and one cytoplasmic helix. To characterize the hydrophobic interface of putative *L*. *stagnalis* RHO, we predicted the hydrophobicity of the of the structure in AlphaFold2 (Fig 9B), depicting the protein/water interface (blue) and the lipid/protein hydrophobic interface (yellow). Consistent with NMR and generalized molecular surface method quantifications of hydrophobicity [62], *L*. *stagnalis* RHO bore a markedly larger lipid/protein hydrophobic interface compared to its protein/water interface. To assess whether *L*. *stagnalis* RHO exhibited more positively- charged residues in the cytoplasmic than the extracellular regions, thus making it conducive to interactions with negatively charged G-protein residues, we assessed the electrostatic potential of this predicted structure (Fig 9C). Indeed, we found that the cytoplasmic side of *L*. *stagnalis* RHO was more positively charged, consistent with properties of vertebrate sensory rhodopsins that interact with G-proteins to trigger the downstream phototransduction pathway [63].

**Fig 10:**
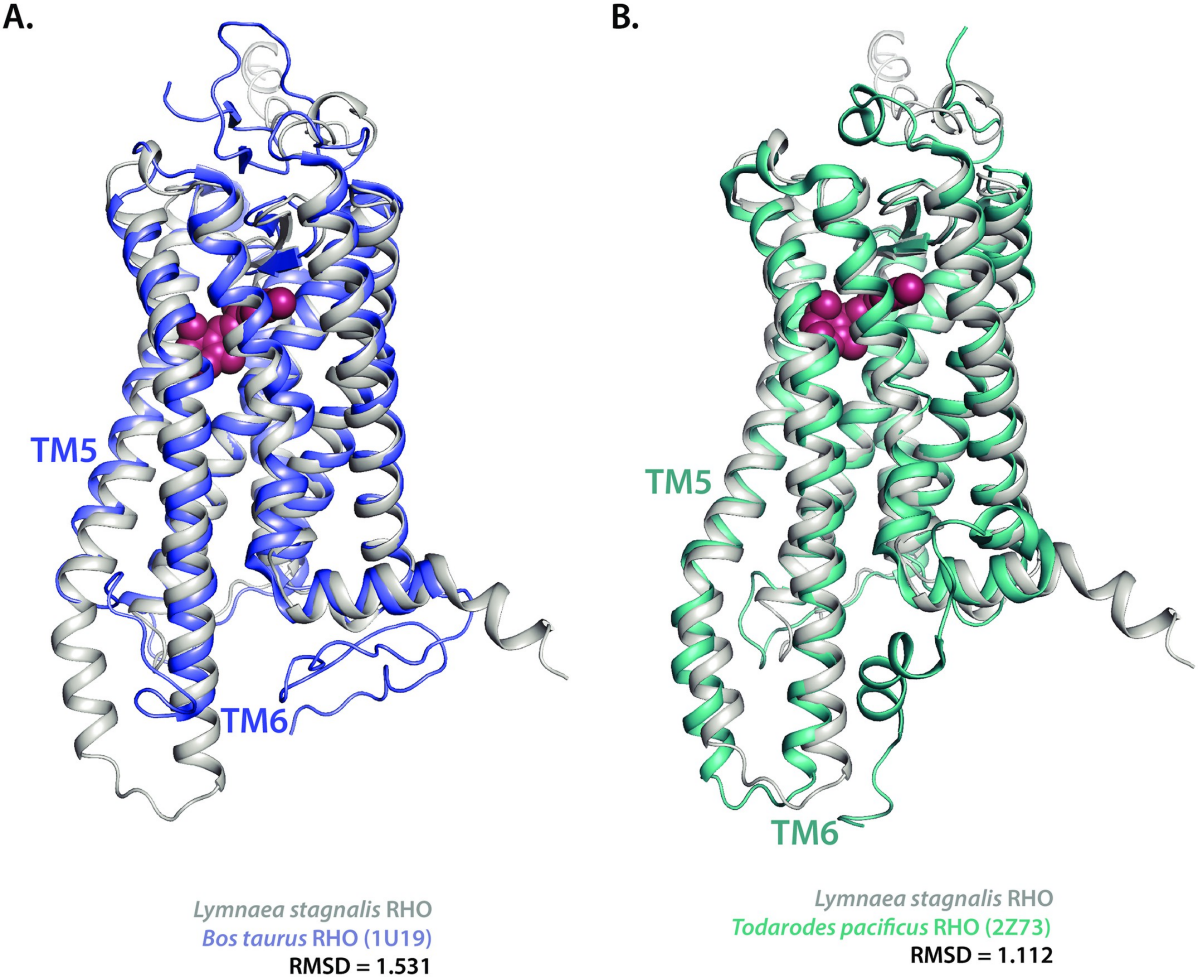
Predicted structural elements of *L*. *stagnalis* rhodopsin depict invertebrate specific structural features. Alphafold2-predicted *L*. *stagnalis* rhodopsin structure aligned to the **(A)** resolved *Bos taurus* rhodopsin (PDB: 1U19) in its native state with bound 11-cis-retinol depicts the shorter transmembrane alpha-helix 5 (TH5) in *B*. *taurus* when compared to *L*. *stagnalis*. The alignment between *L*. *stagnalis* rhodopsin and **(B)** resolved *T*. *pacificus* rhodopsin (PDB: 2Z73) in its inactive state when bound to 11-cis-retinol to *L*. *stagnalis* rhodopsin. Root mean squared deviation (RSMD) values are noted.

Next, we assessed the extent of structural similarities between resolved rhodopsins in their active and active states and our *in silico* predicted 3D *L*. *stagnalis* rhodopsin. First, *L*. *stagnalis* RHO was aligned against the well-characterized bovine inactive rhodopsin (PDB: 1U19) when bound to 11-cis-retinal. We found that both structures have a great deal of overlap in the transmembrane regions (RMSD = 1.531), though a difference in TH5-6 length was notable (Fig 10A), which could be evolutionarily significant to non-Gt-protein binding in invertebrates. Noteworthy is the weak alignment within the linker regions between helices spanning the protein/water interfaces, presumably due to these regions being highly disordered [64].

To better assess these deviations, we plotted the hydrophobicity scores of the *B*. *taurus* and *L*. *stagnalis* alongside *H*. *sapiens* RHO, which itself has few structural deviations from *B*. *taurus*, to depict deviations in conservation (S1A Fig). While *H*. *sapiens* and *B*. *tauru*s rhodopsin showed very similar hydrophobicity scores, *L*. *stagnalis* RHO had a noticeably longer helix 5–6 loop and longer C-terminal region when compared to the vertebrate proteins. Predictions of highly disordered probability confirmed that the N- and C- termini are highly disordered regions of *B*. *taurus* (S1B Fig) and *L*. *stagnalis* (S1C Fig) RHOs. Importantly, noted are select asparagine and serine residues in the N- and C- terminus, respectively, of the *B*. *taurus* rhodopsin that are critical to transducin binding. Given that highly disordered regions often house critical binding sites for protein interactions, future experiments should analyze the evolution and/or conservation of these residues within highly disordered regions to better understand their broad roles in rhodopsin activation and specific interactions with proteins involved in the phototransduction pathways.

To date, few invertebrate rhodopsin 3D structures have been resolved. Thus, given the structural differences between mammalian and *L*. *stagnalis* RHO (Fig 10A), it was imperative to next align *L*. *stagnalis* rhodopsin to a resolved invertebrate mollusk rhodopsin. 3D structural alignment of *L*. *stagnalis* and that of the Japanese squid *Todarodes pacificus* RHO with ll-cis-retinal bound (PDB: 2Z73) [65] show high sequence similarity and similar retinal binding pockets in the overlap between the sequences (RMSD: 1.112; Fig 10B). Notably, the helix 5–6 loops were similar lengthwise, unlike *B*. *taurus* RHO TH5-6 region, which appeared to be much shorter (~10 amino acids). Taken together, this suggests that *L*. *stagnalis* RHO bears some predictable signatures of invertebrate rhodopsins, but at the amino acid level, may differ at critical residues required for GPCR activity and binding with G-coupled proteins.

## Discussion

In this study, we reported the overall structure of *L*. *stagnalis* visual system and described the phylogenetic and phenotypic conservation patterns that may contribute to the phototactic response in *L*. *stagnalis*, where we demonstrated inherent variations in light sensitivity amongst light-sensitive animals. We further explored potentially conserved evolutionary underpinnings of the phototaxis response *in vivo*, while identifying the molecular components of invertebrate phototransduction with a focus on establishing the evolutionary context of *L*. *stagnalis* rhodopsin. Most notably, we determined that despite some differences in the sequence and structure of *L*. *stagnalis* rhodopsin between resolved human, bovine, and squid rhodopsins, *L*. *stagnalis* still exhibits strong phototaxis behaviors. By characterizing phototactic behaviours of *L*. *stagnalis* and confirming that this organism possesses the essential molecular machinery for phototransduction, we have created a replicable foundation upon which to study the visual system in *L*. *stagnalis* and show that the visual system is evolutionarily and functionally conserved but bears unique components within existing pathways, suggesting great potential for future manipulations of this model.

### The structural conservation of visual system components in *L*. *stagnalis* suggest evolutionary conserved mechanisms for photosensitivity

Characterization and summarization of the *L*. *stagnalis* eye structure and primary/secondary projections from the retina to the CNS (Fig 1) provide immense insight into the high-order integration of sensory signals in vestibular/motor activities, though the involvement of these circuits in mediating the animal’s responses to light via phototactic behaviors are unclear. Unlike some opisthobranch gastropods, such as *Hermissenda crassicornis* and *Coryphella rufibranchialis*, who evolved specialized optic ganglia that are likely the crucial site for the integration of first-order projections from the retina and an evolutionary adaptation [9, 10], the organization of the *L*. *stagnalis* visual system is more structurally similar to that of mammals, where visuo-sensory information is detected in the retina, whose projections go directly to the CNS via the optic nerve [13, 66], presumably for integration and mediation of photosensation to produce light-guided locomotive outcomes.

Accordingly, secondary retinal connections include projections to the pedal ganglia, which contain locomotory serotonergic neurons, while *in vivo* injury of the peripheral nerves of the pedal ganglia results in locomotory deficits [67], suggesting that photosensitive locomotion may be dependent on secondary retinal projections to the pedal ganglia acting as the ‘motor centre’ for visuo-mediated locomotory output in the central ring ganglia [68]. While we detailed the eye and central ring ganglia connectivity, the statocyst’s contributions to visual processing behaviors in *L*. *stagnalis* is notable given the importance of this gravitometric organ throughout Mollusca and may indicate that these projections to/from the statocyst are integrated with the vestibular response [68]. Indeed, the statocyst of the sea slug *Hermissenda* helps to govern the snail’s orientation and is light-responsive [9] and is the relay centre for communication between the photoreceptor cells of the retina and hair cells in a unidirectional manner [11]. Hair cells of the statocyst are sensitive to light and turbulence and are driven by ocular photoreceptors which form projections to caudal hair cells [69], which may drive snails’ orientation to light and serve to integrate signal for secondary projections throughout the ganglia network.

Second order retinal projections from the statocyst hair cells and cerebral ganglia traverse to the ipsilateral pedal ganglia, which itself is a relay center for both sensory and motor information and is implicated in locomotion through the presence of motor neurons and the pedal nerve fibres to locomotory muscles [70]. Importantly, light-sensitive retinal neurons found in the retinal pigment layer have been proposed to integrate sensory signals and regulate light sensitivity through efferent projections [66], such as those to the statocyst, which may indicate the propensity of these projections to be integrated into the vestibular response. Given that mollusks are thought to have evolved over 550 million years ago [71], it is important to appreciate the contralateral and ipsilateral pathways by which visual information is transmitted to the central ring ganglia in *L*. *stagnalis* [72], generating intricate contralateral and ipsilateral connections between the visual and vestibular systems [73], presumably for higher-order integration of visual stimuli elsewhere in the central ring ganglia, leading to further binocular integration of visual stimuli as demonstrated in the terrestrial slug *Limax valentianus* [74]. Future works may explore the role of the statocyst in phototaxis and locomotory outcome in naïve and statocyst injured animal to best assess the integration of visual information into the vestibular-locomotory response.

Importantly, within the *L*. *stagnalis* retina, we identified photo-sensitive pigment granules are organized in the retinal pigment layer (Fig 2-A2, A3), consistent with *L*. *stagnalis* pigment structures characterized in previous works [13], as well as in other gastropod mollusks [40, 75, 76]. Through our electron microscopy work, we show distinct layering of the *L*. *stagnalis* retina (Fig 3A and 3B) and the apparent presence of two retinal cells within the microvilli and pigment layers, dubbed pigment cells and photoreceptor sensory cells. In previous studies, two neuronal cell types have been identified in the *L*. *stagnalis* retina: photoreceptor cells and optic ganglion cells [77]. While there have been efforts to characterize the *L*. *stagnalis* eye through light and electron microscopy have been done previously [8, 39, 78], we provide a detailed image of the pigment and sensory cells of the *L*. *stagnalis* retina (Fig 3C), where the later may correspond to the neuronal cell types previously described [66, 77, 78]. The ultrastructure of the sensory cells greatly resembles those detailed previously in Patellogastropoda [79] and is consistent with *Viviparus viviparus* light micrographs [80] that speculated that both microvillar and ciliary projection in the retina of this mollusk allow for a greater photon-absorbing photoreceptive surface, and subsequently, light-directed locomotion. While it is unclear whether *L*. *stagnalis* sensory cells bear both cilia and microvilli projections in our preparations, it is reasonable to assume that the strong conservation of retinal structure makes it so that *L*. *stagnalis* bears both cilia and microvilli that may operate under differing light transduction mechanisms, given the presence of molecules in both vertebrate and invertebrate phototransduction pathways.

The presence of pigment granules is notable, given that retinal pigment epithelium in mammals is thought to have evolved from the granular retinal pigment layer found in the invertebrate retinal pigment epithelium [81], and thus photosensitive pigment granules in *L*. *stagnalis* may maintain retinal homeostasis similarly to mammals [82]. Our present results demonstrated the location of the stained nuclei adjacent to the retinal pigment layer (Fig 2-B2) is consistent with previous literature showing that light-sensitive type A photoreceptors located within the pigment layer of the retina terminate within the neural layer of the retina, though axons of the type T photoreceptors cell bodies, located in the pigment layer, make up the optic nerve bundle and terminate the cerebral ganglia and statocyst [11, 66]. In previous reports, three optic cell types have been identified in the *L*. *stagnalis* retina—two rhabdomeric photoreceptor cells, dubbed Type A and Type T photoreceptors, and optic ganglion cells [39, 66, 83]. Consistent with other gastropod mollusks, these two photoreceptor cells generate electrical signals in response to light which are accompanied by an elevation in intracellular calcium [25], the latter of which is common to ocular photoreceptor function and physiology across the animal kingdom [84].

Given the absence of rhabdomeric membranes in vertebrates [85], mollusk photoreceptor cells may be evolutionary precursors to photosensitive retinal ganglion cells in vertebrates [86], and may be an interesting caveat for retinal ganglion cell evolution. On the other hand, visual system integration in *L*. *stagnalis* CNS is complicated by the presence of non-ocular photoreceptors that drive locomotion independent of ocular photoreception [4, 12, 87]. Indeed, retinal connectivity to the tentacles occurs through the first-order retinal projections through the cerebral commissure, implicating the tentacles in light sensitivity. The presence of rhodopsin-positive cells in the skin adjacent to the eye (Fig 2-C1), coupled with a positive phototaxis response in snails lacking eyes and tentacles [12] may suggest that the dermal photoreceptors play a role in phototaxis behaviors in *L*. *stagnalis*, perhaps as a compensatory mechanism when ocular photoreception is hindered. Thus, future research should seek to explore the relationship between ocular and dermal photoreceptors to determine the extent of their contributions toward the positive-phototaxis response.

### Machine learning models and tracking snails phototaxis behavior provide the basis to establish *L*. *stagnalis* as a critical model for vision sciences and vision orientated locomotory behavior

In this study we developed a new phototaxis neurobehavioral test where snails locomoted on their foot toward a presentation of strong focal light in a dark box arena, thus producing a positive phototaxis response and reducing peripheral light-driven thigmotactic responses (Fig 4A). The peripheral light-occluding design of our arena represents an improvement on previous assessments of *L*. *stagnalis* phototaxis, as it is consistent with recent methods to assess positive phototaxis in larval zebrafish (*Danio rerio*), where reducing peripheral light-driven thigmotactic responses that may occlude focal light-driven phototactic responses were accounted for [88]. As well, the location of the focal light in the arena is an important consideration to this design of this model and allows us to extrapolate key information on an animal’s locomotory preference toward light. In this phototaxis arena, the fixed focal light source was placed opposite of the *L*. *stagnalis* starting end of the arena, to extrapolate a given animals trajectory length when it was placed at a standard distance from the target light. Though future considerations for augmentation of this arena design may be to account for phototaxis preference [89] and spectral sensitivity of *L*. *stagnalis* eyes [87], it is important to consider the effects of different wavelengths of the light on the light-sensitive locomotory parameters observed in this study.

To date, most assessments of locomotory behaviour in the *L*. *stagnalis* phototaxis response have made qualitative observations and quantification of parameters associated with this locomotory behavior have been limited. As well, while our group has previously explored quantitative measurements of locomotion [67] and the visual response [90], though there was a pressing need for a neurobehavioral test that assessed snail locomotory response to a visual stimulus in a high-throughput and reliable manner to better understand mollusk photoreception. Thus, we aimed to provide a robust quantitative characterization of locomotory behaviour patterns and parameters associated with these patterns, and discovered that light-sensitive animals (i.e., those that exhibited positive phototaxis) demonstrated either weak photosensitivity or strong photosensitivity (Fig 5A and 5B). Strong photosensitive animals exhibited greater mean trajectory lengths during the focal light phases of testing compared to weak photosensitive animals (Fig 5C), suggesting that strong photosensitive animals display stronger purposeful movement in response to strong focal light. Early studies of phototaxis in the mollusk *Hermissenda* found that ~80% of animals tested exhibited positive phototaxis behaviors in different phototaxis arenas, spending more time in the focal light area at increased light intensities when compared to red light and dim light conditions [91]. This is consistent with our findings that a majority of our tested cohort (n = 23; 76.7%) only displayed strong locomotion towards the focal light area during the strong focal light condition, and not under the dark conditions. The diversity in phototaxis response is evident through our model, where some animals (n = 6; 20.7%) had weaker phototaxis responses but were more active in the focal light than in the dark. By critically computing quantitative parameters of the phototaxis response using DeepLabCut, representing the first ever use of a deep machine learning (ML) pose-estimation model for quantification of locomotory parameters in a mollusk vision model, we were able to identify different patterns of phototaxis in *L*. *stagnalis*. To our knowledge, analysis of this kind has seldom been done before in gastropod mollusks, paving the way for future research to apply this deep-learning model to behavioral paradigms in *L*. *stagnalis* phototaxis behavior after ocular injury and with known CPGs involved in the aerial respiratory behavior [92–95], to further extrapolate key patterns of snail behavior.

Given the robustness and high-throughput nature of our neurobehavioral test and downstream analysis, this model has a high degree of potential to be used to evaluate effects of environmental or physical manipulations on behavioural phenotypic outcome, such as the effects of retinal injury on phototaxis. In zebrafish, retinal laser injury leads to retinal disorganization, focal lesions, and a marked decrease in retinal cell abundance [96], thereby affecting light-sensitivity. The effects of such a manipulation in *L*. *stagnalis* on phototaxis could therefore be tested using the model established in the current study, to gain insights into how photoreceptor loss or disruption drives pathological manifestations in higher animals, such as the onset of macular degeneration in humans [97] given the the similarities in visual system structure. Thus, it is critical to explore injury to the pigmented retinal layer in *L*. *stagnalis* to assess whether pigmented photoreceptors cells drive phototaxis. While early work in *L*. *stagnalis* found that eye enucleation and optic nerve severing seldom influenced positive phototaxis behavior output, blinded snails may have oriented less well toward the light source [12]. Thus, our established model provides the opportunity to robustly identify patterns of behavior and correlations between the structural and behavioral changes following photoreceptor insult.

### Insights into the evolutionary conservation of molecular identities involve involved in vision and light sensitivity in *L*. *stagnalis* provides insight on the molecular processes governing rhodopsin-dependent phototransduction

Through this work, we have conducted phylogenetic surveys of vision and phototransduction components to speculate on the molecular underpinnings of photosensitive, positive phototactic behaviour in this model. Taking advantage of recent advances in the quality of the *L*. *stagnalis* transcriptome, we quantified TPM expression data for molecular identities critical to phototransduction (Fig 6B), namely rhodopsin, in the *L*. *stagnalis* CNS. Of note, the mollusk CNS houses photo-sensitive neurons that may use rhodopsin mediated signalling pathways to detect and react to light and response by exciting or inhibiting neuron activity [54], which we speculate may be why phototransduction pathway signatures are present in the *L*. *stagnalis* CNS. Previous research has shown that *L*. *stagnalis* photoreceptors, clade with photopigment granules, bear rhodopsin and arrestin immunofluorescence signatures exclusively in the rhabdomeric membranes of the retina and at the surface of the skin in dermal photoreceptors [23]. Given that rhodopsin is a light-sensitive G-protein coupled receptor protein that triggers visual phototransduction in light-sensitive cells [17, 98], while arrestin functions to terminate the light response [99, 100], it is reasonable to assume that some signalling components of the phototransduction pathway are conserved between *L*. *stagnalis* and vertebrates. Indeed, in *L*. *valentianus*, a single β-arrestin identified in the CNS was found to be localized to rhabdomeric membranes of photosensory neurons in the retina and may have served as a precursor to the evolution of visual arrestins in later diverging animals [101]. More broadly, critical identities in the visual phototransduction pathway, such as various visual opsins, cyclic nucleotide gated (CNG) ion channels, and high/low light responsive visual phosphodiesterase (PDE6), appear to all be well conserved in bilaterian animals [102, 103], and together with the identification of rhodopsin- and arrestin-positive cells in *L*. *stagnalis* sensory tissues [23], suggests that mollusks like *L*. *stagnalis* have the molecular machinery with which to exhibit phototransduction.

The low level of rhodopsin mRNA in the snail CNS relative to β-actin, GAPDH, and β-tubulin homologs (Fig 6B) is consistent with mammalian findings, where rhodopsin is primarily localized to photosensitive tissues of the retina (i.e., photoreceptors) with little rhodopsin protein expression in the cerebral cortex (Human Protein Atlas proteinatlas.org; [104, 105)]. Importantly, however, characterization of the light activated chromatophore expansion in the cephalopod mollusk *Octopus bimaculoides* found their skin to be light sensitive and driven by an r-opsin phototransduction cascade, while subsequent research in *Octopus vulgaris* would implicate the rhodopsin kinase gene, GRK1, in extra-ocular light perception despite it being most abundantly expressed in the retina [106]. While we identified that GRK1 has low expression in the *L*. *stagnalis* CNS (0.4118 ± 0.0877) (Fig 6B), it appears that conservation of light sensing molecules and their expression in non-ocular tissues across Metazoa is important to understanding the evolution of light sensing behaviors to inform on the most early diverging mechanisms governing vision that may be conserved in higher organisms. Indeed, rhodopsin has been found to localize to mammalian keratinocytes and melanocytes in mammals, implicating this protein in photobiomodulation [107]. Previous studies into the molecular identities of the phototransduction pathway in *L*. *stagnalis* identified dermal phototransduction to be initiated by the rhabdometric opsins (r-opsins) and proposed to have phosphoinositol signalling cascade [108]. As the phototransduction signalling process in *L*. *stagnalis* remains unclear, especially given the presence of both vertebrate and invertebrate phototransduction homologs in the *L*. *stagnalis* CNS transcriptome, future research may explore whether conserved molecular identities involved in phototransduction are abundantly expressed elsewhere in *L*. *stagnalis*, such as in the skin/dermis, to speculate of the conserved molecular pathways driving perception to light. Given the existence of two phototransduction pathway molecules, it is reasonable to speculate that the large repertoire of phototransduction pathway molecules identified in this study are involved in rhodopsin-independent opsin-mediated pathways in *L*. *stagnalis* photosensory tissues, given that other gastropod mollusks exhibit multiple opsins that are responsible for integrating visual light stimuli [40]. Nevertheless, our transcriptomic analysis of *L*. *stagnalis* CNS to inform on visual opsin genes and other phototransduction-related genes provides a preliminary framework towards understanding and differentiating the mechanisms of phototransduction in the photoreceptors of the skin and retina.

Using Kyte-Doolittle hydrophobicity predictions, we confirmed that *L*. *stagnalis* RHO bears seven hydrophobic transmembrane helices (Fig 7A), which was further corroborated by protein alignments (Fig 8) and AlphaFold2 structural predictions (Fig 9A), though resolution of RHO is imperative to best deduce structure-function relationships between this *L*. *stagnalis* RHO and identified phototransduction pathway homologs. Nonetheless, through phylogenetic inferences, we showed that *L*. *stagnalis* rhodopsin may not group phylogenetically with other invertebrate Gq-coupled rhodopsins (Fig 7B), suggesting that *L*. *stagnalis* may possess atypical RHO functionality when compared to other Gq-coupled rhodopsins and may induce a phototransduction pathway differently. Typically, rhodopsins belong to one of two phylogenetically distinct subgroups: vertebrate rhodopsins that activate cGMP-specific phosphodiesterase via the G-protein transducin (Gt-coupled), and invertebrate rhodopsins that activate phospholipase C via a Gq-type G-protein (Gq-coupled) [109]. However, the invertebrate scallop *M*. *yessoensis* has a unique rhodopsin that is localized to the retina and bears strong sequence similarly to mammals but employs a Go-signalling cascade (Go-coupled) [22]. While future phylogenetic assessments should include all visual opsin clades to better understand the phylogenetic placement of *L*. *stagnalis* RHO, the findings that *L*. *stagnalis* rhodopsin does not confidently group with Gt-, Gq-, or Go-coupled receptors in our are suggestive of *L*. *stagnalis* rhodopsin using a unique G-protein signalling cascade.

We reported that the predicted *L*. *stagnalis* RHO has a longer, though less hydrophobic, helix 5–6 loop (TH5-6) (S1 Fig), which is consistent with the resolved squid rhodopsin structure. Given that the TH5-6 region is involved in photoactivation of the channel and shifts to produce a binding pocket for transducin alpha-subunit in vertebrates, these differences in the TH5-6 length and critical amino acids could be a result of the unique binding needs of an alternative G-coupled protein. Considering that highly disordered regions house critical binding sites for protein interactions [110], future experiments should analyze the evolution and/or conservation of these residues within highly disordered regions to better understand their broad roles in rhodopsin activation and specific interactions with proteins involved in the phototransduction pathways.

## Conclusion

In this comprehensive study, we delved into the intricate visual system of the pulmonated gastropod mollusk *L*. *stagnalis* and explore the evolutionary and molecular aspects of its phototactic response. Through a detailed analysis of *L*. *stagnalis*’ visual apparatus, we identified structural and molecular components that could be contributing to its light-sensitive behavior. This study unveiled the presence of specialized rhodopsin-positive photoreceptor cells and pigment granules within the retina, enforcing the evolutionary conservation of these features across mollusks. Additionally, a novel neurobehavioral test was developed using DeepLabCut, enabling in-depth quantitative assessment of phototactic responses in *L*. *stagnalis*. Surprisingly, despite distinct differences in the rhodopsin protein sequence and structure compared to other invertebrates, *L*. *stagnalis* exhibited robust phototaxis behaviors. The findings not only provided valuable insights into the unique visual mechanisms of *L*. *stagnalis* but also highlighted the conservation of essential phototransduction processes, paving the way for further exploration of this model organism in vision-related research. This study’s significance lies in its establishment of *L*. *stagnalis* as a vital model for understanding vision sciences, offering a foundation for future investigations into the molecular and evolutionary aspects of photosensitivity and phototaxis behaviors.

## Supporting information

S1 File Phototaxis neurobehavioral protocol and DeepLabCut processing methods. (PDF)

S1 Fig Analysis of predicted disordered regions within *Bos taurus* and *Lymnaea stagnalis* rhodopsin reveals similar patterns of disordered probability. **(A)** Kyte-Dootlittle plot depicting transmembrane (>0) and cytoplasmic/extracellular (<0) spanning regions of H. sapiens rhodopsin (NP_000530.1), *B*. *taurus* rhodopsin (NP_001014890.1) and *L*. *stagnalis* rhodopsin proteins reveals length differences between the mammalian and *L*. *stagnalis* cytoplasmic helix 5–6 loop domains. PrDOS predicted regions of increased protein disorder, namely the N-terminus, the cytoplasmic ‘bridge’ between helix 5–6 and the C-terminus are indicated for **(B)** *B*. *taurus* rhodopsin (NP_001 014890.1) and **(C)** *L*. *stagnalis* rhodopsin homolog. Amino acids critical to light-sensing abilities (Asn2 and Asn15) and to arrestin binding (Ser334, Ser338 and Ser343) are highlighted within the N-terminus and C-terminus, respectively. The threshold for disordered region predictions (blue line) and the predicted disordered possibility for each protein of interest (dashed grey line) are indicated.
(TIF)
