# Supplementary material for: Functional characterization of optic photoreception in Lymnaea stagnalis
Source: PLoS One. 2024 Nov 12;19(11):e0313407. doi: 10.1371/journal.pone.0313407 (PMC11556747; doi:10.1371/journal.pone.0313407)
Supplement: S1 File — (PDF) [file pone.0313407.s001.pdf]

Phototaxis analysis supplementary methods

Arena Design

A clear, rectangular glass arena (25.4L x 10.2W x 10.2H centimetres) was inserted into a black paperboard box (approximately 32.0L x 10.4W x 10.4H centimetres) to occlude any peripheral light entering the arena. A NOVAFLEX Fiber Optic Illuminator (World Precision Instruments) was placed at the far end of the arena, projecting uniform illumination (power level 5) through a hole in the black paperboard box and into the arena. The arena consisting of an open rectangular glass container set in an open black cardboard box to create a contained arena for assessing locomotion under different lighting conditions and a pinhole at the extreme end of the arena to introduce strong focal light via a fibre optic light source. The dimensions of the focal light zone, the area featuring the strongest focal light luminance, was determined by a single-blind labeller, and provided as a reference 'bounding box' of light during analysis (Figure 3A). Locomotion responses were recorded aerially on infrared RaspberryPi camera, allowing for high resolution video acquisition of animal movements during the dark and focal light conditions (Supplementary File 1; MP4). Snails' responses were observed during two phases of testing—dark phase and focal light phase (Figure 3B), where responses these phases were the focus of this analysis to highlight differences in locomotive response, if any, in the presence or absence of focal light presentation. While locomotive outcomes in response to light have been studied before in molluscs, the onset of markerless pose-estimation of animal behaviors for object recognition offers advantages for neural network training and data extrapolation. Using the established DeepLabCut pipeline, 50 frames per video acquired were extracted and labelled at a snail's head and various points of its shell train a deep neural network on such that the model could accurately predict the movement of the animal over approximately 60,000 video frames (See Supplementary File 2). To quantify behaviour, positional output data for all frames was obtained, and various parameters of the trajectory were analyzed in R (Figure 3D), such as trajectory length, trajectory speed, and straightness index of the trajectory travelled.

Recording Light Conditions

Within the arena (shown below, birds-eye view), the luminosity measured at 22.7°C are as follows, with readings taken at positions 1-4:

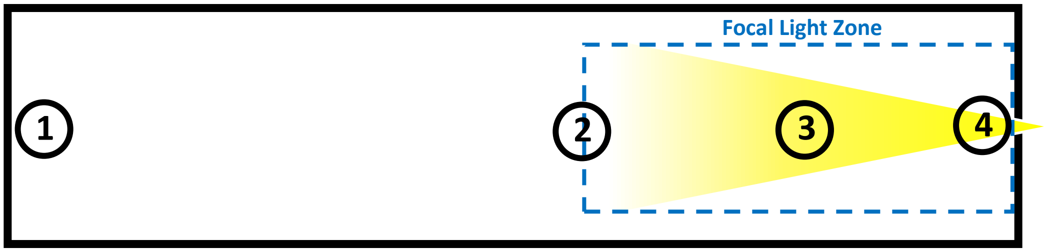

Phase	Minimum Luminosity	Maximum Luminosity
Dark Phase (Position 2)	0.0	0.0
Focal Light Phase (Position1)	316	322
Focal Light Zone (Position 2)	1020	1085
Focal Light Zone (Position 3)	1733	1853
Focal Light Zone (Position 4)	4530	4812

DeepLabCut post-processing

Behavioural trajectory analysis
Behavioural analysis of DeepLabCut positional output was carried out in R (version 4.2.2). Portions of the recording window where the camera's view of the animal was obscured were excluded from analysis, as were frames where the pixel likelihood as computed by DeepLabCut was less than or equal to 0.8. The apex of the animal was used to estimate its position as its labeling was the most consistent in all frames.

Parameters extracted from DeepLabCut trajectories were analyzed for each acclimation, dark, and focal light phase using both base R, Tidyverse packages (version 2.0.0), and trajr (version 1.4.0)[1].

1. *Trajectory length (trajlength)*

   Trajectory length was calculated along the entire trajectory using TrajLength() from trajr, as the sum of step-wise distances between points in the trajectory.

2. *Mean speed (speed_mean)*

   The mean speed was calculated as the trajectory length as defined by TrajLength() from trajr by the trajectory duration as defined by TrajDuration() from trajr, thus yielding:

$$Speed = \frac{trajlength\ (pixels)}{duration\ (s)}$$

3. *Time in light in minutes (time_in_light_min)*

   The time spent in the focal light area was defined as the difference between the maximum and minimum times at which the animal's y-coordinate was greater than or equal to the bottom y-coordinate of the focal light bounding box:

```
time_in_light = max(time[y >= y_min]) - min(time[y >= y_min])
```

   Note that this therefore means that multiple entries into the focal light bounding box as well as the x-coordinates of the bounding box were not considered. The resulting time in seconds was divided by 60 to yield time in minutes.
