# Supplementary figures and images for: Functional characterization of optic photoreception in Lymnaea stagnalis
Source: PLoS One. 2024 Nov 12;19(11):e0313407. doi: 10.1371/journal.pone.0313407 (PMC11556747; doi:10.1371/journal.pone.0313407)

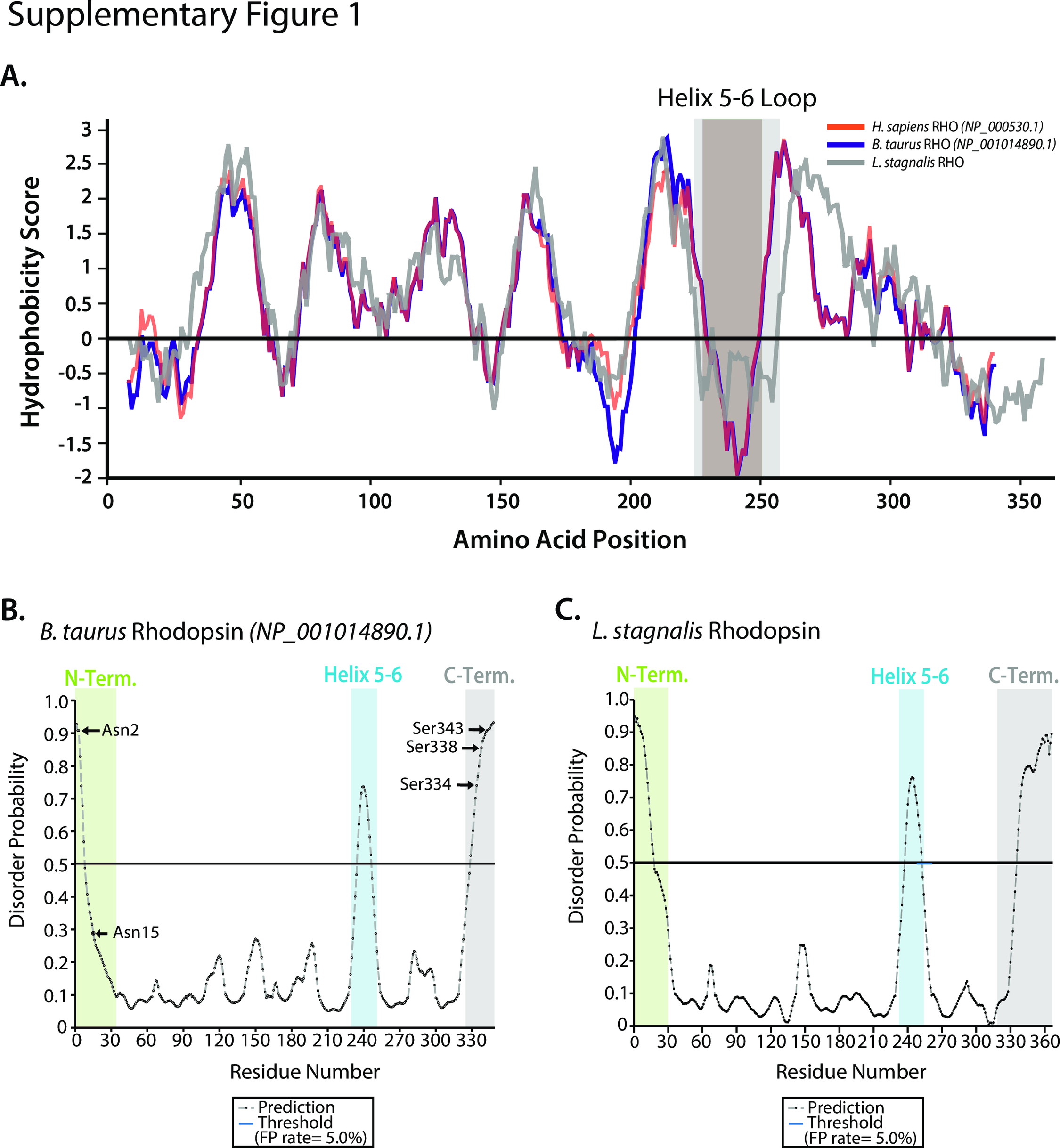

Supplement: S1 Fig — (A) Kyte-Dootlittle plot depicting transmembrane (>0) and cytoplasmic/extracellular (<0) spanning regions of H. sapiens rhodopsin (NP_000530.1), B. taurus rhodopsin (NP_001014890.1) and L. stagnalis rhodopsin proteins reveals length differences between the mammalian and L. stagnalis cytoplasmic helix 5–6 loop domains. PrDOS predicted regions of increased protein disorder, namely the N-terminus, the cytoplasmic ‘bridge’ between helix 5–6 and the C-terminus are indicated for (B) B. taurus rhodopsin (NP_001 014890.1) and (C) L. stagnalis rhodopsin homolog. Amino acids critical to light-sensing abilities (Asn2 and Asn15) and to arrestin binding (Ser334, Ser338 and Ser343) are highlighted within the N-terminus and C-terminus, respectively. The threshold for disordered region predictions (blue line) and the predicted disordered possibility for each protein of interest (dashed grey line) are indicated. (TIF) [file pone.0313407.s002.tif]
